# Dietary Carbohydrate and Diverse Health Outcomes: Umbrella Review of 30 Systematic Reviews and Meta-Analyses of 281 Observational Studies

**DOI:** 10.3389/fnut.2021.670411

**Published:** 2021-04-29

**Authors:** Ya-Shu Liu, Qi-Jun Wu, Jia-Le Lv, Yu-Ting Jiang, Hui Sun, Yang Xia, Qing Chang, Yu-Hong Zhao

**Affiliations:** ^1^Department of Clinical Epidemiology, Shengjing Hospital of China Medical University, Shenyang, China; ^2^Clinical Research Center, Shengjing Hospital of China Medical University, Shenyang, China

**Keywords:** carbohydrate, dietary, health outcomes, meta-analysis, umbrella review

## Abstract

**Background and Aims:** The associations between dietary carbohydrate and diverse health outcomes remain controversial and confusing. To summarize the existing evidence of the association between dietary carbohydrate intake and diverse health outcomes and to evaluate the credibility of these sources of evidence. We performed this umbrella review of evidence from meta-analyses of observational studies.

**Methods:** PubMed, Embase, Web of Science databases, and manual screening of references up to July 2020 were searched. Systematic reviews with meta-analyses of observational studies in humans investigating the association between dietary carbohydrate intake and multiple health outcomes were identified. We assessed the evidence levels by using summary effect sizes, 95% prediction intervals, between-study heterogeneity, evidence of small-study effects, and evidence of excess significance bias for each meta-analysis.

**Results:** We included 43 meta-analyses of observational research studies with 23 health outcomes, including cancer (*n* = 26), mortality (*n* = 4), metabolic diseases (*n* = 4), digestive system outcomes (*n* = 3), and other outcomes [coronary heart disease (*n* = 2), stroke (*n* = 1), Parkinson's disease (*n* = 1), and bone fracture (*n* = 2)]. This umbrella review summarized 281 individual studies with 13,164,365 participants. Highly suggestive evidence of an association between dietary carbohydrate intake and metabolic syndrome was observed with adjusted summary odds ratio of 1.25 [95% confidence interval (CI) 1.15–1.37]. The suggestive evidences were observed in associations of carbohydrate consumption with esophageal adenocarcinoma (0.57, 95% CI = 0.42–0.78) and all-cause mortality (adjusted summary hazard ratio 1.19, 95% CI = 1.09–1.30).

**Conclusions:** Despite the fact that numerous systematic reviews and meta-analyses have explored the relationship between carbohydrate intake and diverse health outcomes, there is no convincing evidence of a clear role of carbohydrate intake. However, there is highly suggestive evidence suggested carbohydrate intake is associated with high risk of metabolic syndrome, suggestive evidence found its association with increased risk of all-cause mortality and decreased risk of esophageal adenocarcinoma.

**Systematic Review Registration:** CRD42020197424.

## Introduction

Dietary carbohydrates constitute a specific group of substances with a range of chemical, physical, and physiological properties, which serve as the main and preferable source of body energy (1, 2). Carbohydrates are classified into available carbohydrates and resistant carbohydrates based on their digestion and absorption by the digestive system (3). Available carbohydrates can be absorbed in the small intestine and provide energy to important tissues, such as the brain, red blood cells, and the developing fetus (3, 4). Resistant carbohydrate, such as prebiotics and resistant starch, may influence the activity of intestinal microbiota and other physiological function (1, 3).

Historically, in terms of energy supplied to glucose-dependent tissues, such as brain, consumption of increased amounts of carbohydrate may have provided a substantial evolutionary advantage (4). The importance of dietary carbohydrate intake for disease prevention has been stressed by health organizations and programs such as the American Cancer Society and the National Cholesterol Education Program (NCEP) (5). The intake of carbohydrate recommended by the NCEP is 50–60% of the total calories (6). However, dietary carbohydrate intake has been an anathema in the public view due to different reasons including worries about obesity and adverse effects of carbohydrates on the level of high-density lipoprotein and triglycerides (5). In fact, these seemingly contradictory concepts are due to the different effects of various types of carbohydrates on health. A previous umbrella review found that there is convincing evidence of an inverse association between whole grains (contain fiber, vitamins, minerals, and phytochemicals with antioxidant properties) consumption and risk of type 2 diabetes and colorectal cancer (7). Nevertheless, refined carbohydrates, such as white rice and noodles, might reflect poor food quality and confer a chronically high glycemic load that can lead to negative metabolic consequences and other adverse health outcomes (8). In the last decade, numerous epidemiologic studies have shown that dietary carbohydrate intake is potentially linked to increasing risk of many health outcomes including total mortality (8), endometrial cancer (9), colorectal cancer (10), coronary heart disease (11), type 2 diabetes (12), metabolic syndrome (13), cortical and nuclear cataract (14), which are of great important to public health and societal cost. However, the findings from these studies are, to some extent, controversial and confusing as substantial heterogeneity and inherent study biases (such as residual confounding and selective reporting of positive results). For example, the association between dietary carbohydrate intake and mortality has not been unified (8, 15, 16).

To the best of our knowledge, there has been no attempt to comprehensively summaries the studies addressing the association between dietary carbohydrates intake and multiple health outcomes. Previous efforts to systematically appraise the evidence on dietary carbohydrate have been focused on single disease endpoints. Hence, we performed the first umbrella review of published meta-analyses and systematic reviews. The aim of the present study is to investigate the breadth and strength of the existing evidence by systematically assessing the quality of the studies in order to identify potential biases and highlight those with the strongest evidence of medical evidence.

## Methods

### Umbrella Review Methods

Umbrella review is a method that synthesizes a large number of existing systematic reviews and/or meta-analyses on risk factors, rather than performing these systematic reviews from the beginning. We conducted an umbrella review, i.e., a comprehensive and systematic search, to organize, and evaluate the existing evidence of dietary carbohydrate intake and diverse health outcomes from multiple systematic reviews and meta-analyses (17). The protocol of the study was registered on PROSPERO (CRD42020197424). This study was performed following the Meta-analyses Of Observational Studies in Epidemiology (MOOSE) guidelines (Supplementary Table 1) (18).

### Literature Search

Two independent investigators (Y-SL and J-LL) comprehensively searched the published literature using the PubMed, Embase, and Web of Science databases from inception to July 10, 2020 for systematic reviews or meta-analyses of observational studies that evaluated the evidence regarding the effects of dietary carbohydrate on health. The key words used in the search strategy were “carbohydrate” or “carbohydrates,” and “meta” or “meta-analysis” or “systematic review.” The detailed search strategy is shown in Supplementary Table 2. No language restrictions were considered for the selection of eligible studies for this review. Furthermore, we conducted a manual search of the reference lists of the retrieved articles. A third investigator (Q-JW) arbitrated any differences that could not be resolved by consensus. Only data from published papers were included, and the study authors were not contacted.

### Study Selection and Exclusion Criteria

Articles were eligible if they were meta-analyses and had been conducted systematically. We included only meta-analyses or systematic reviews of observational studies (cohort studies, case-control studies, and cross-sectional studies) in humans. Meta-analyses were included when they summarized any combination of relative risks, odds ratios, relative rates, or hazard ratios from studies investigating the association between dietary carbohydrate and any health-related outcome (e.g., cardiovascular disease, cancer, death, obesity or overweight, diabetes, and metabolic diseases).

No randomized controlled trials related to our research question were found. We excluded studies of genetic polymorphisms of carbohydrate metabolism. Systematic reviews without a quantitative synthesis, meta-analyses based on individual data without a systematic review, or systematic reviews of ecological studies with no individual data were excluded. Meta-analyses or systematic reviews that did not present study specific data [risk estimates, 95% confidence intervals (CIs), or the number of events, controls, or total sample size] were also excluded.

Separate meta-analyses for different eligible outcomes within individual articles were assessed separately. When more than one meta-analysis presented overlapping datasets on the same outcome, only the meta-analysis with the largest dataset was retained for the main analysis. All the selection and exclusion procedures were carried out by two independent investigators (Y-SL and Q-JW).

### Data Abstraction

Data extraction was performed independently by two investigators (Y-SL and Y-TJ) using a custom-made data extraction form. Disagreements were re-evaluated by a third investigator (Q-JW). When a meta-analysis or systematic review reported both summarized results and results divided according to subgroups, the summarized results were preferred, since they were derived from a larger sample. The following key study characteristics were abstracted from each systematic review and meta-analysis: (1) first author, (2) publication year, (3) journal name, (4) study design, (5) number of studies included, (6) total number of cases and participants, (7) outcome(s) of interest, and (8) type of effect metric.

For primary studies from each systematic review and meta-analysis included, the following key study characteristics were abstracted: (1) first author, (2) publication year, (3) study design, (4) study population, (5) outcome(s) of interest investigated, (6) unit of exposure comparison, (7) methods of ascertainment of dietary carbohydrate intake (e.g., food-frequency questionnaire and 24-h recall), (8) type of comparison (high vs. low analysis or dose-response analysis), (9) total number of cases and/or controls, (10) maximally adjusted risk estimates and 95% CIs, and (11) the effect of dose-response relation. If a risk factor was examined in more than one comparison, we extracted the data from the comparison with the largest number of component studies.

### Assessment of the Methodological Quality of the Included Studies

The methodological quality of the meta-analyses included was assessed using the validated AMSTAR (A Measurement Tool to Assess Systematic Reviews) instrument. AMSTAR measures 11 items that allow a comprehensive evaluation of systematic reviews (19). AMSTAR has been shown to be a reliable and valid tool for the quality assessment of systematic reviews and meta-analyses of both interventional and observational research (20, 21), which includes search quality ratings, analysis, and transparency of a meta-analysis. The answers include “yes,” “no,” “cannot answer,” and “not applicable” for every question. For each “yes” response, participants score one point, and the maximum score is 11. The methodological quality was categorized into high quality, moderate quality, and low quality based on an overall score of at least 8 points, 4–7 points, and 3 points or less, respectively (20, 22).

### Statistical Analyses

For each exposure and outcome pair, we calculated the summary effect and the 95% CI using fixed and random effects methods, respectively, to test the stability and reliability of associations between dietary carbohydrate and health outcomes. Furthermore, 95% prediction intervals (95% PIs) for the summary random effects estimates were used to show true effects for 95% of the summarized studies or similar (exchangeable) studies that might be conducted in the future (23, 24). For the largest study of each meta-analysis, we estimated the standard error (SE) of the effect size and examined whether the SE was <0.10. In a study with a SE of <0.10, the difference between the effect estimates and the upper or lower 95% CI was <0.20 (i.e., this uncertainty is lower than what is considered a small effect size) (25).

We used the *I*^2^ statistic as an estimate of proportion of variance reflecting true differences in effect size. Values exceeding 50% or 75% were considered to represent large or very large heterogeneity, respectively (26). We assessed evidence for small study effects using the regression asymmetry test proposed by Egger and colleagues (27). A *P-*value of ≤ 0.10 in the regression asymmetry test with a more conservative effect in the largest study was considered evidence for small-study effects bias (28).

We applied the excess significance test to investigate whether the observed number of studies (O) with nominally significant results (“positive” studies, *P* < 0.05) was different from the expected number of significant results (E). The detailed description of this method is described in a previous study (28). Briefly, the expected number of studies with significant results is calculated in each meta-analysis via the sum of the statistical power estimates for each component study (29). Since the true effect size for any meta-analysis is not known, we estimated the power of each component study using the effect size of the largest study (smallest SE) in a meta-analysis (29). The statistical power of each study was calculated using an algorithm from a non-central *t* distribution (30). Excess significance for single meta-analyses was defined at *P* < 0.10. The comparison of observed vs. expected values was performed separately for each meta-analysis, and was also extended to groups, including many meta-analyses, after summing the observed and expected values from each meta-analysis. As described elsewhere, the number of expected “positive” (i.e., significant data sets) studies can be compared with the observed number of significant studies through a chi-square-based test (29). The larger the difference between observed and expected values, the higher the excess of significance bias.

All analyses were performed using STATA software, version 15 (StataCorp, College Station, TX).

### Credibility Assessment

We categorized the strength of the evidence of dietary carbohydrate intake for outcomes into convincing, highly suggestive, suggestive, weak evidence, or non-significant associations according to the following criteria (31).

1. The evidence was defined as convincing when the *P*-value of the random-effects model was smaller than 10^−6^, the meta-analysis included more than 1,000 cases or more than 20,000 participants for continuous outcomes, if the largest component study in the meta-analysis reported a significant result (*P* < 0.05), if the 95% PIs excluded the null hypothesis, if the *I*^2^ statistic for heterogeneity was <50%, if there was no evidence of small study effects (*P* > 0.10), and if excess significance bias (*P* > 0.10) was indicated.
2. The evidence was defined as highly suggestive if the *P*-value for the random-effects model was <10^−6^, if the meta-analysis included more than 1,000 cases or more than 20,000 participants for continuous outcomes, and if the largest component study reported a significant result.
3. The evidence was defined as suggestive if the *P*-value for random-effects was < 10^−3^, or if there were more than 1,000 cases or more than 20,000 participants for continuous outcomes.
4. The evidence was defined as weak if the *P*-value for significant associations was <0.05.
5. We used the ‘non-significant associations' classification if all association tests yielded a *P*-value > 0.05.

## Results

**Figure 1:**
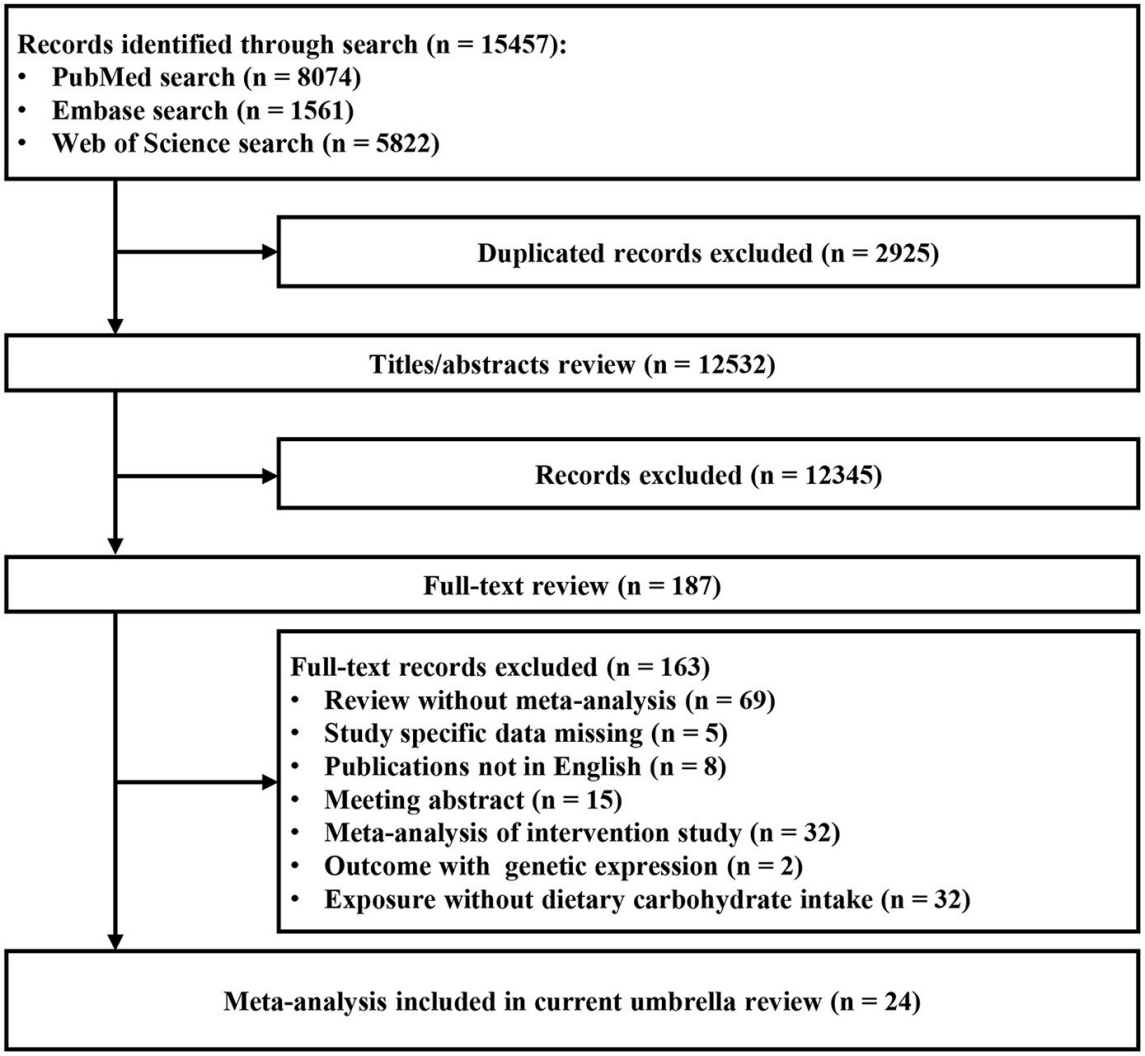
Flowchart of selection of studies for inclusion in umbrella review on dietary carbohydrate intake and health outcomes.

Of the 12,532 articles (excluding duplicates) initially identified in three databases, 12,345 articles were excluded after title and abstract review. Finally, 24 eligible articles were identified (8–13, 15, 32–48). The search yielded 43 meta-analyses after full-text screening (Figure 1). After data abstraction, 30 of the 43 meta-analyses were selected for the main analysis for systematically assessing the quality of existing evidence. The characteristics of the 24 articles are shown in Supplementary Table 3. The list of excluded studies and the exclusion grounds during the process of full-text review are shown in Supplementary Table 4.

### Characteristic of the Meta-Analysis

In all 43 meta-analyses identified via full-text screening, more than one published meta-analysis was examined for 23 outcomes, including cancer [breast cancer (*n* = 1 meta-analysis), colorectal cancer (*n* = 4), digestive system cancers (*n* = 2), endometrial cancer (*n* = 3), esophageal adenocarcinoma (*n* = 2), esophageal cancer (*n* = 3), esophageal squamous cell carcinoma (*n* = 2), gastric cancer (*n* = 2), liver cancer (*n* = 1), pancreatic cancer (*n* = 3), prostate cancer (*n* =3)], mortality [all-cause mortality (*n* = 2), specific-cause mortality (*n* = 2)], metabolic diseases [type 2 diabetes (*n* = 3), metabolic syndrome (*n* = 2)], digestive system outcomes [ulcerative colitis (*n* = 1), Crohn's disease (*n* = 1), inflammatory bowel diseases (*n* = 1)], other outcomes [coronary heart disease (*n* = 2), stroke (*n* = 1), Parkinson's disease (*n* = 1), and bone fracture (*n* = 2)]. The percentage of outcomes per category is shown in Supplementary Figure 1. The characteristics, quantitative synthesis, and credibility assessment of evidence of all eligible meta-analyses are shown in Supplementary Tables 5, 6, respectively.

After retaining the largest dataset for the main analysis, there were 30 meta-analyses summarizing 281 individual studies. The median number of primary studies in each meta-analysis was eight (range: 4–22), the median number of cases was 3,600 (range: 388–69,164), and the median number of participants was 309,923 (range: 1,344–2,666,588). The number of cases was >1,000 in 27 meta-analyses (90.0%), three meta-analyses included <1,000 cases (outcomes: Crohn's disease, ulcerative colitis, and liver cancer).

### Summary Effect Size

Of the 30 meta-analyses, summary random effects estimates were significant (*P* ≤ 0.05) in 9 (30.0%), whereas summary fixed effects were significant in 11 meta-analyses (36.7%). At a stricter threshold of *P* < 0.001, 3 (10.0%) and 6 (20.0%) meta-analyses produced significant summary results using the random and fixed effects models, respectively. At *P* < 10^−6^, 1 (3.3%) and 4 (13.3%) meta-analyses were significant by the random and fixed effects models, respectively (Table 1). Moreover, the largest study showed statistically significant results in 4 (13.3%) meta-analyses (Table 2). The effects of the largest studies were more conservative than the summary effects of the meta-analysis in 13 of the 30 (43.3%) meta-analyses.

**Table 1:** The characteristics and quantitative synthesis of the eligible meta-analyses reporting dietary carbohydrate intake relation to multiple outcomes.

**Outcomes**	**Individual study**	**No. of primary studies**	**No. of cases/participants**	**Comparison**	**Summary relative risk (95% CI)**		**Random *P*-value^†^**	**Fixed *P*-value^‡^**
					**Random effects**	**Fixed effects**		
**Mortality**								
All-cause mortality	Sara B Seidelmann, 2018	5	30942/287644	low vs. moderate	1.19 (1.09–1.30)	1.14 (1.09–1.20)	1.456 × 10^−4^	1.785 × 10^−8^
Stroke and specific–cause mortality^*^	Xianlei Cai, 2015	6	1831/170348	high vs. low, dose–response	1.12 (0.92–1.36)	1.12 (0.93–1.35)	0.258	0.236
All–cause and specific–cause mortality^§^	Dale S. Hardy, 2020	5	4191/110411	high vs. low	1.04 (0.91–1.19)	1.04 (0.91–1.19)	0.561	0.561
**Cancer**								
Breast cancer	Sabrina Schlesinger, 2017	11	30201/885890	dose–response	1.00 (0.96–1.05)	1.00 (0.98–1.02)	0.955	0.953
Colorectal cancer	D. Aune, 2012 (a)	9	9246/783980	dose-response	0.95 (0.85–1.07)	0.93 (0.87–1.00)	0.403	0.041
	Jian Huang, 2017	16	11400/843184	high vs. low	1.08 (0.93–1.26)	1.01 (0.93–1.10)	0.308	0.791
Digestive system cancers	Xianlei Cai, 2019	20	11594/2666588	high vs. low	1.01 (0.93–1.10)	1.00 (0.93–1.07)	0.784	0.972
Endometrial cancer	Alireza Sadeghi, 2019	6	3998/490255	high vs. low	1.08 (0.87–1.33)	1.09 (0.98–1.22)	0.486	0.110
	Alireza Sadeghi, 2019	6	3998/490255	dose-response	1.02 (0.98–1.05)	1.01 (0.99–1.03)	0.307	0.230
Esophageal adenocarcinoma	Fei Xuan, 2020	10	1798/9459	high vs. low	0.57 (0.42–0.78)	0.64 (0.54–0.77)	3.788 × 10^−4^	7.549 × 10^−7^
Esophageal cancer	Xianlei Cai,2019	9	1842/440440	high vs. low	0.75 (0.53–1.06)	0.74 (0.62–0.89)	0.099	0.001
Esophageal squamous cell carcinoma	Kondwani-Joseph Banda, 2020	8	1218/5974	high vs. low	0.63 (0.45–0.90)	0.70 (0.57–0.86)	0.012	4.871 × 10^−4^
Gastric cancer	Xianlei Cai, 2019	12	2355/111631	high vs. low	0.84 (0.58–1.22)	0.96 (0.83–1.12)	0.368	0.607
Liver cancer	Xianlei Cai, 2019	6	674/655527	high vs. low	1.04 (0.83–1.30)	1.04 (0.83–1.30)	0.720	0.720
Pancreatic cancer	D. Aune, 2012 (b)	9	3202/1112404	high vs. low	1.00 (0.86–1.15)	1.00 (0.88–1.14)	0.962	0.999
	D. Aune, 2012 (b)	9	3202/1112404	dose-response	0.97 (0.81–1.16)	0.99 (0.86–1.14)	0.713	0.857
Prostate cancer	Lai lai Fan, 2018	22	11573/98583	high vs. low	1.11 (0.98–1.26)	1.17 (1.10–1.23)	0.101	4.920 × 10^−8^
**Metabolic diseases**								
Type 2 diabetes	Greenwood DC, 2013	8	18403/336161	dose-response	0.97 (0.90–1.06)	0.99 (0.95–1.02)	0.514	0.484
	Amani Alhazmi, 2014	8	11536/488969	high vs. low, dose-response	1.11 (1.01–1.22)	1.13 (1.05–1.21)	0.035	0.001
Metabolic syndrome	Yashu Liu, 2019	18	69164/283150	high vs. low	1.25 (1.15–1.37)	1.24 (1.18–1.29)	5.262 × 10^−7^	2.477 × 10^−21^
	Yashu Liu, 2019	10	12081/45729	dose-response	1.02 (1.00–1.05)	1.01 (1.00–1.02)	0.023	4.523 × 10^−4^
**Digestive system outcomes**								
Ulcerative colitis	Fan Wang, 2016	5	540/2075	dose-response	1.01 (0.99–1.02)	1.00 (0.99–1.01)	0.440	0.423
Crohn's disease	Lirong Zeng, 2017	4	388/1344	dose-response	0.99 (0.98–1.00)	0.99 (0.98–1.00)	0.166	0.166
Inflammatory bowel diseases	Zhongqin Jin, 2018	15	1361/332202	high vs. low	1.09 (0.82–1.46)	1.08 (0.93–1.26)	0.555	0.315
**Other outcomes**								
Coronary heart disease	Geoffrey Livesey, 2019	6	2507/228209	dose-response	1.65 (1.19–2.29)	1.50 (1.21–1.85)	0.002	1.840 × 10^−4^
	Dale S. Hardy, 2020	11	15316/464491	high vs. low	1.08 (1.00–1.16)	1.07 (1.01–1.13)	0.045	0.016
Stroke	Geoffrey Livesey, 2019	8	7283/394020	high vs. low	1.11 (0.94–1.31)	1.11 (0.95–1.30)	0.228	0.197
Parkinson's disease	Aimin Wang, 2015	8	1482/232869	high vs. low	1.24 (1.05–1.48)	1.24 (1.05–1.48)	0.014	0.014
Bone fracture	H.Mozaffari, 2020	5	1635/38828	high vs. low	1.24 (0.84–1.83)	0.96 (0.82–1.13)	0.276	0.645
	H.Mozaffari, 2020	6	1765/41341	dose-response	1.00 (0.94–1.05)	1.00 (0.99–1.01)	0.896	0.661

*CI, confidence interval*.

* *Specific-cause mortality included hemorrhagic and ischemic stroke mortality*.

§ *Specific-cause mortality included Type 2 Diabetes mortality, coronary heart disease mortality, and stroke mortality*.

† *P-value of summary random effects estimate*.

‡ *P-value of summary fixed effects estimate*.

*All statistical tests were two-sided*.

**Table 2:** Credibility assessment of evidence for the meta-analyses reporting association of dietary carbohydrate intake relation to multiple outcomes.

**Outcomes**	**Individual study**	**Features used for credibility assessment of evidence**						**Evidence classification**
		**Sample size^†^**	**Statistical significance^‡^**	**Largest study Significance**	**95% prediction interval**	**Estimate of heterogeneity^#^**	**Small-study effect/excess significant bias**	
**Mortality**								
All-cause mortality	Sara B Seidelmann, 2018	> 1,000	<10^−3^	> 0.05	Including the null value	Large	Small-study effects	Suggestive
Stroke and specific-cause mortality^*^	Xianlei Cai, 2015	> 1,000	> 0.05	> 0.05	Including the null value	Not large	Neither	No association
All-cause and specific-cause mortality^§^	Dale S. Hardy, 2020	> 1,000	> 0.05	> 0.05	Including the null value	Not large	Neither	No association
**Cancer**								
Breast cancer	Sabrina Schlesinger, 2017	> 1000	> 0.05	> 0.05	Including the null value	Large	Neither	No association
Colorectal cancer	D. Aune, 2012 (a)	> 1000	> 0.05	<0.05	Including the null value	Large	Neither	No association
	Jian Huang, 2017	> 1000	> 0.05	> 0.05	Including the null value	Large	Small-study effects	No association
Digestive system cancers	Xianlei Cai, 2019	> 1000	> 0.05	> 0.05	Including the null value	Not large	Neither	No association
Endometrial cancer	Alireza Sadeghi, 2019	> 1000	> 0.05	> 0.05	Including the null value	Large	Neither	No association
	Alireza Sadeghi, 2019	> 1000	> 0.05	> 0.05	Including the null value	Large	Neither	No association
Esophageal adenocarcinoma	Fei Xuan, 2020	> 1000	<10^−3^	> 0.05	Including the null value	Large	Neither	Suggestive
Esophageal cancer	Xianlei Cai,2019	> 1000	> 0.05	> 0.05	Including the null value	Large	Neither	No association
Esophageal squamous cell carcinoma	Kondwani Joseph Banda, 2020	> 1000	<0.05	> 0.05	Including the null value	Large	Neither	Weak
Gastric cancer	Xianlei Cai, 2019	> 1000	> 0.05	> 0.05	Including the null value	Very large	Neither	No association
Liver cancer	Xianlei Cai, 2019	<1000	> 0.05	> 0.05	Including the null value	Not large	Neither	No association
Pancreatic cancer	D. Aune, 2012 (b)	> 1000	> 0.05	> 0.05	Including the null value	Not large	Neither	No association
	D. Aune, 2012 (b)	> 1000	> 0.05	> 0.05	Including the null value	Not large	Neither	No association
Prostate cancer	Lai lai Fan, 2018	> 1000	> 0.05	<0.05	Including the null value	Large	Neither	No association
**Metabolic diseases**								
Type 2 Diabetes	Greenwood DC, 2013	> 1000	> 0.05	<0.05	Including the null value	Very large	Neither	No association
	Amani Alhazmi, 2014	> 1000	<0.05	<0.05	Including the null value	Not large	Neither	Weak
Metabolic syndrome	Yashu Liu, 2019	> 1000	<10^−6^	<0.05	Including the null value	Large	Excess significance bias	Highly suggestive
	Yashu Liu, 2019	> 1000	<0.05	> 0.05	Including the null value	Very large	Neither	Weak
**Digestive system outcomes**								
Ulcerative colitis	Fan Wang, 2016	<1000	> 0.05	> 0.05	Including the null value	Not large	Neither	No association
Crohn's disease	Lirong Zeng, 2017	<1000	> 0.05	> 0.05	Including the null value	Not large	Neither	No association
Inflammatory bowel diseases	Zhongqin Jin, 2018	> 1000	> 0.05	> 0.05	Including the null value	Not large	Neither	No association
**Other outcomes**								
Coronary heart disease	Geoffrey Livesey, 2019	> 1000	<0.05	> 0.05	Including the null value	Not large	Neither	Weak
	Dale S. Hardy, 2020	> 1000	<0.05	> 0.05	Including the null value	Not large	Neither	Weak
Stroke	Geoffrey Livesey, 2019	> 1000	> 0.05	> 0.05	Including the null value	Not large	Neither	No association
Parkinson's disease	Aimin Wang, 2015	> 1000	<0.05	> 0.05	Including the null value	Not large	Neither	Weak
Bone fracture	H.Mozaffari, 2020	> 1000	> 0.05	> 0.05	Including the null value	Large	Neither	No association
	H.Mozaffari, 2020	> 1000	> 0.05	> 0.05	Including the null value	Large	Neither	No association

* *Specific-cause mortality included hemorrhagic and ischemic stroke mortality*.

§ *Specific-cause mortality included Type 2 Diabetes mortality, CHD mortality, and stroke mortality*.

† *Number of cases*.

‡ *P-value under the random-effects model*.

# *Heterogeneity was categorized as not large (I^2^ <50%), large (I^2^ ≥ 50% but I^2^ ≤ 75%), and very large (I^2^ > 75%)*.

### Heterogeneity Between Studies

There was large heterogeneity in 13 (43.3%) meta-analyses and very large heterogeneity in three (10.0%) meta-analyses. The meta-analyses with very large heterogeneity included outcomes such as type 2 diabetes, gastric cancer, and metabolic syndrome. In addition, uncertainty of the summary effects was further assessed by calculating 95% PIs. The 95% PIs of all meta-analyses included the null value (Table 2).

### Small Study Effects and Excess Significance

According to the Egger's test results, two (6.7%) meta-analyses (colorectal cancer and all-cause mortality) showed small study effects, and a more conservative effect was found in the largest studies. For the remaining 28 (93.3%) meta-analyses, no evidence was found for small-study effects and comparison between the summary random effects estimate and the point estimate of the largest study. Excess significance was present at *P* < 0.1 and O > E. As a result, only one (3.3%) meta-analysis tested positive in the excess significance test (Table 2).

### Strength of Evidence

Based on the findings, the meta-analyses with nominally significant summary results were categorized into convincing, highly suggestive, suggestive, or weak evidence (Table 3). No meta-analysis presented convincing evidence, one (3.3%) presented highly suggestive evidence of the association between an increase in dietary carbohydrate intake and a higher risk of metabolic syndrome, and two (6.7%) presented suggestive evidence for three outcomes. In these studies, dietary carbohydrate intake was found to be associated with decreased risk of esophageal adenocarcinoma, and increased risk of all-cause mortality. Six (20.0%) meta-analyses with five outcomes presented weak evidence. The evidence reported for a negative effect of dietary carbohydrate intake on four different outcomes (coronary heart disease, type 2 diabetes, metabolic syndrome, and Parkinson's disease) was considered weak, as was the positive association between dietary carbohydrate intake and esophageal squamous cell carcinoma (Table 3).

**Table 3:** Summary of evidence grading for studies reporting dietary carbohydrate intake relation to multiple outcomes.

**Evidence category**	Criteria^*^	**Health benefits**	**Health risks**
Convincing (*n* = 0^†^)	Statistical significance at *P* < 10^−6^; > 1,000 cases (or > 20,000 participants for continuous outcomes); The largest component study reported a significant effect (*P* < 0.05); The 95% prediction interval excluded the null; No large heterogeneity (I^2^ < 50%); No evidence of small-study effect (*P* > 0.10) and excess significance bias (*P* > 0.10).	None	None
Highly suggestive (*n* = 1^†^)	Statistical significance at *P* < 10^−6^; > 1,000 cases (or > 20,000 participants for continuous outcomes); The largest component study reported a significant effect (*P* < 0.05).	None	Metabolic syndrome
Suggestive (*n* =2^†^)	Statistical significance at *P* < 10^−3^; > 1,000 cases (or > 20,000 participants for continuous outcomes);	Esophageal adenocarcinoma	All-cause mortality
Weak (*n* = 6^†^)	Statistical significance at *P* < 0.05	Esophageal squamous cell carcinoma	Coronary heart disease Type 2 diabetes Parkinson's disease Metabolic syndrome

* *The criteria of no association: statistical significance at P > 0.05 (number of studies = 29)*.

† *The number of studies reporting dietary carbohydrate intake relation to multiple outcomes*.

### Methodological Quality

Overall scores of AMSTAR for each eligible article are shown in Supplementary Figure 2, and single items are summarized in Supplementary Table 7. The AMSTAR score ranged from 5 to 10. Eight (33.3%) studies were considered to be of high quality and 16 (66.7%) of moderate quality. No study fell in the low or critically low quality category. In general, the main flaws in these eligible articles were that the study design was not established before the conduct of the review, the gray literature was not accounted for in the literature search, and the list of excluded studies was not provided.

## Discussion

### Principal Findings

Our umbrella review provides an overview and appraisal of meta-analyses on the link between dietary carbohydrate intake and multiple health outcomes. Overall, our review examined the association of dietary carbohydrate intake with 23 outcomes, including cancer, mortality, metabolic diseases, digestive system diseases, and other outcomes. There was no association supported by convincing evidence with strong epidemiological credibility. There is evidence highly suggestive of the correlation between dietary carbohydrate intake and increased risk of metabolic syndrome. We observed suggestive evidence for the associations of carbohydrate consumption with decreased risk of esophageal adenocarcinoma and increased risk of all-cause mortality.

There is weak evidence of the dose-response correlation between dietary carbohydrate intake and increased risk of metabolic syndrome and coronary heart disease. A meta-analysis including 284,638 participants indicated that carbohydrate intake and prevalence of metabolic syndrome, with a 2.6% increase in the risk of metabolic syndrome per 5% energy intake from carbohydrates. In addition, a meta-analysis of prospective cohort studies suggested that per 98 g/d carbohydrate increment was associated with 65% increased risk of coronary heart disease.

### Comparison With Other Studies and Possible Explanations

Evidence from 3 cohort and 15 case-control studies supported a risk effect of carbohydrate consumption for metabolic syndrome. So far, there are no reference values of carbohydrate intake for the prevention and treatment of metabolic syndrome. The European Food Information Council indicated that the European Food Safety Authority has provided a reference range of carbohydrate daily intake between 45 and 60% combined with reduced fat and saturated fat intake to improve metabolic risk factors for chronic disease (49). Moreover, the highly suggestive evidence was consistent with a meta-analysis of randomized controlled trials. This meta-analysis estimated the long-term (6-month or longer) effect of low-carbohydrate diets on metabolic risk factors, and indicated an association of low-carbohydrate diets with loss of weight and improvement in metabolic risk factors (50). In addition, a previous study suggested that metabolic regulation of insulin gene expression enables cells to maintain adequate stores of intracellular insulin to sustain the secretory demand. Glucose is the major physiological regulator of insulin gene expression (51) and this may partly explain the association between dietary carbohydrate intake and level of blood glucose, which is a component of metabolic syndrome. Notably, our results showed that a higher intake of carbohydrate was weakly associated with a higher risk of type 2 diabetes. Moreover, the dose-response analyses revealed a significant association between carbohydrate consumption and risk of type 2 diabetes. Similarly, low-quality evidence of an association between total carbohydrates intake and type 2 Diabetes was found in another umbrella review (22). In addition, we found evidence of excess significance bias for the association between carbohydrate intake and metabolic syndrome, which resulted in a relative excess of reported statistically significant results as compared to what would be expected in a body of evidence (29).

We found suggestive evidence of an association between higher dietary carbohydrate intake and lower risk of esophageal adenocarcinoma. The key mediators associating dietary and lifestyle factors with carcinogenesis have been reported to be hyperinsulinemia, insulin-like growth factor, and insulin resistance (52, 53). Elevated insulin levels could inhibit the development and progression of many types of cancer by suppressing insulin-like growth factor-binding protein or by increasing the production of insulin receptors (54–56). Hence, dietary carbohydrate intake could decrease the risk of oesophageal adenocarcinoma by raising the levels of insulin. Moreover, a higher intake of carbohydrate could reflect a higher consumption of plant-based foods (especially fruits and vegetables), which has been confirmed to have an inverse relationship with oesophageal cancer (57). However, there was no credible evidence of an association between carbohydrate intake and other cancer types.

Suggestive evidence was also found for the association between carbohydrate consumption and risk of all-cause mortality. This finding was consistent with that from a large perspective cohort study in 135,335 individuals from 18 countries, with a median follow-up of 7.4 years (58). Notably, another meta-analysis pooled the effect size of different geographic regions to investigate the association between carbohydrate consumption and mortality. This meta-analysis suggested an insignificant association between the intake of carbohydrates and mortality in Europe and Asia (15). Another prospective cohort study using data from the US National Health and Nutrition Examination Survey from 1999 to 2014 from 37,233 Adult, also indicated differences in the association of low-carbohydrate with mortality among non-Hispanic whites and other populations in the US (59). Moreover, a study showed that a long- standing refined grain-based diet might have contributed to trigger the evolution of biological adaptations to mitigate the side effects of diets (60). Further evidence from a genome-wide meta-analysis of observational studies revealed an association between common genetic variants and macronutrient intake (61). Thus, it is reasonable to hypothesize that genetic factors may influence the association between dietary carbohydrate intake and mortality. In future studies, the genetic variability in carbohydrate metabolism should be considered. Meanwhile, more and higher quality research will aid the development of reference values for carbohydrate intake for different ethnic groups and disease-state populations.

### Strength and Limitations

This study is the first umbrella review to provide a systematic and comprehensive summary of the published literature to explore the role of carbohydrates in human health-related outcomes. This review may also help investigators to judge the relative importance of carbohydrates for various health outcomes. Moreover, we evaluated the methodological quality using AMSTAR, which offered good evidence of validity and reliability. Beyond assessing the quality of studies, we explored the extent of bias and heterogeneity among the included meta-analyses. Furthermore, we identified the low quality evidence of the association between dietary carbohydrate intake and multiple outcomes to provide directions for future research.

The findings of our work should also be interpreted in light of its limitations. Although we used systematic methods that included a robust search strategy, in many meta-analyses the authors have stated their assumption but included insufficient information to allow us to judge the suitability of the pooling. Thus, these meta-analyses with insufficient information were excluded from our review. Moreover, the umbrella review is a method of synthesizing existing evidence, which depends on the selection of the estimates from each primary study and its representation in the meta-analysis. Hence, the included individual studies with poor quality outcomes need to be interpreted with caution. Nevertheless, none of the meta-analyses included in our umbrella review had low or critically low methodological quality. Thus, bias due to quality of the studies had little effect on the results. Furthermore, this umbrella review had an observational study design, and hence bias from reverse causality and recall bias cannot be avoided. In addition, although the most important confounders were adjusted for in most of the primary studies, residual confounding cannot be completely ruled out.

Significantly, the differences of amount of comparison in each primary study may cause inaccurate evaluations of summary effects. These inevitable differences should attribute to the meta-analyses which pooled the risk estimates from the highest vs. lowest category of exposure. For example, one meta-analysis included two studies which both compared the highest quartile with the lowest quartile intake of dietary carbohydrate. However, the highest and lowest quartiles of these two studies were completely different (303 vs. 242 g/day and 162 vs. 114 g/day) (Supplementary Table 3) (33). Dose-response studies are less susceptible to such bias, but only ten Dose-response Meta-analysis Were included in the present umbrella review. Therefore, this result should be interpreted with caution. For this reason, future primary studies with dose-response analysis are needed to interpret the summary estimates more reasonably in evidence-based medicine.

## Conclusions

Our study found highly suggestive evidence of the association between dietary carbohydrate intake and increased risk of metabolic syndrome, and suggestive evidence of the association of carbohydrate consumption with increased risk of mortality and decreased risk of esophageal adenocarcinoma. Notably, the evidence of the associations between carbohydrate intake and other health outcomes were weak or even non-existent. Thus, the enthusiasm of low carbohydrate diet and the anathema of low carbohydrate food should be reconsidered. Furthermore, the large heterogeneity of dietary assessment methods and the inadequacies related to the study design suggest the need for recommendations for guiding future interventions to be sufficiently powered to detect clinical outcomes. Future randomized controlled trials in large sample sizes are needed to confirm these observational findings and to study the effects of different carbohydrate subtypes.

## Data Availability Statement

The original contributions presented in the study are included in the article/Supplementary Material, further inquiries can be directed to the corresponding author/s.

## Author Contributions

Q-JW and Y-HZ contributed to the study design. Y-SL and J-LL conducted the literature search. Y-SL, Q-JW, and Y-TJ extracted the data and conducted the analyses. Y-SL, HS, YX, and QC wrote the first draft of the manuscript and edited the manuscript. All authors contributed to the article and approved the submitted version.

## Conflict of Interest

The authors declare that the research was conducted in the absence of any commercial or financial relationships that could be construed as a potential conflict of interest.

## Abbreviations

NCEP: National Cholesterol Education Program
AMSTAR: A Measurement Tool to Assess Systematic Reviews
SE: standard error
CIs: confidence intervals
PIs: prediction intervals.
