# Supplementary material for: Dietary Carbohydrate and Diverse Health Outcomes: Umbrella Review of 30 Systematic Reviews and Meta-Analyses of 281 Observational Studies
Source: Front Nutr. 2021 Apr 29;8:670411. doi: 10.3389/fnut.2021.670411 (PMC8116488; doi:10.3389/fnut.2021.670411)
Supplement: Supplementary file 1 [file Table_1.DOCX]

**Supplementary Material**

Supplementary table 1. MOOSE Checklist for Meta-analyses of Observational Studies

| **Item No** | **Recommendation** | **Reported on Page No** |
| --- | --- | --- |
| Reporting of background should include | | |
| 1 | Problem definition | 4 |
| 2 | Hypothesis statement | 4-5 |
| 3 | Description of study outcome(s) | 4-5 |
| 4 | Type of exposure or intervention used | 4 |
| 5 | Type of study designs used | 5 |
| 6 | Study population | 4-5 |
| Reporting of search strategy should include | | |
| 7 | Qualifications of searchers (eg, librarians and investigators) | 6 |
| 8 | Search strategy, including time period included in the synthesis and key words | 6 and supplementary table 1 |
| 9 | Effort to include all available studies, including contact with authors | 6 |
| 10 | Databases and registries searched | 6 |
| 11 | Search software used, name and version, including special features used (eg, explosion) | 6 |
| 12 | Use of hand searching (eg, reference lists of obtained articles) | 6 |
| 13 | List of citations located and those excluded, including justification | supplementary tables 3 |
| 14 | Method of addressing articles published in languages other than English | 7 |
| 15 | Method of handling abstracts and unpublished studies | 6 |
| 16 | Description of any contact with authors | 6 |
| Reporting of methods should include | | |
| 17 | Description of relevance or appropriateness of studies assembled for assessing the hypothesis to be tested | 7-9 |
| 18 | Rationale for the selection and coding of data (eg, sound clinical principles or convenience) | 7-8 |
| 19 | Documentation of how data were classified and coded (eg, multiple raters, blinding and interrater reliability) | not applicable |
| 20 | Assessment of confounding (eg, comparability of cases and controls in studies where appropriate) | 8-9 |
| 21 | Assessment of study quality, including blinding of quality assessors, stratification or regression on possible predictors of study results | 8-9 |
| 22 | Assessment of heterogeneity | 9-10 |
| 23 | Description of statistical methods (eg, complete description of fixed or random effects models, justification of whether the chosen models account for predictors of study results, dose-response models, or cumulative meta-analysis) in sufficient detail to be replicated | 9-10 |
| 24 | Provision of appropriate tables and graphics | Table 1 |

| **Item No** | **Recommendation** | **Reported on Page No** |
| --- | --- | --- |
| Reporting of results should include | | |
| 25 | Graphic summarizing individual study estimates and overall estimate | Table 2, 3 |
| 26 | Table giving descriptive information for each study included | supplementary tables 2 |
| 27 | Results of sensitivity testing (eg, subgroup analysis) | not applicable |
| 28 | Indication of statistical uncertainty of findings | Table 2, 3 |
| Reporting of discussion should include | | |
| 29 | Quantitative assessment of bias (eg, publication bias) | 14 |
| 30 | Justification for exclusion (eg, exclusion of non-English language citations) | 17-18 |
| 31 | Assessment of quality of included studies | 18 |
| Reporting of conclusions should include | | |
| 32 | Consideration of alternative explanations for observed results | 18-19 |
| 33 | Generalization of the conclusions (ie, appropriate for the data presented and within the domain of the literature review) | 18-19 |
| 34 | Guidelines for future research | 19 |
| 35 | Disclosure of funding source | 19-20 |

*From*: Stroup DF, Berlin JA, Morton SC, et al, for the Meta-analysis Of Observational Studies in Epidemiology (MOOSE) Group. Meta-analysis of Observational Studies in Epidemiology. A Proposal for Reporting. *JAMA*. 2000;283(15):2008-2012. doi: 10.1001/jama.283.15.2008.

Transcribed from the original paper within the NEUROSURGERY® Editorial Office, Atlanta, GA, United Sates. August 2012.

Supplementary table 2. Search strategy used in the umbrella review.

| Database | Literature search strategy |
| --- | --- |
| PubMed | #1: carbohydrate OR carbohydrates  #2: meta [Title/Abstract] OR meta analysis [Title/Abstract] OR systematic review [Title/Abstract]  #3: #1 AND #2  Search equation: ((meta[Title/Abstract]) OR (meta analysis[Title/Abstract]) OR (systematic review[Title/Abstract])) AND ((carbohydrates) OR (carbohydrate)) |
| Web of Science | #1: TS= (carbohydrate OR carbohydrates)  #2: TI= (meta OR meta analysis OR systematic review)  #3: #1 AND #2  Search equation: TS= (carbohydrate OR carbohydrates) AND TI= (meta OR meta analysis OR systematic review) |
| EMBASE | #1: “carbohydrate” OR “carbohydrates”  #2: “meta” ab,ti OR “meta analysis” ab,ti OR “systematic review” ab,ti  #3: #1 AND #2  Search equation: (carbohydrate OR carbohydrates) AND (meta:ab,ti OR 'meta analysis':ab,ti OR 'systematic review':ab,ti) |

Supplementary table 3. Description of the eligible articles reporting dietary carbohydrate intake relation to multiple outcomes.

| Individual study | N of studies | Study design | Outcome | Type of effect metric | Exposure assessment | Category and amount of comparison in each primary study |
| --- | --- | --- | --- | --- | --- | --- |
| Yao Ye 2016 ^40^ | 26 | cohort study, case-control study | gastric cancer | RR | NA | NA |
| Kondwani Joseph Banda 2020 ^35^ | 26 | case-control study | esophageal cancer, esophageal adenocarcinoma, esophageal squamous cell carcinoma | RR, OR | HHHQ; FFQ; Self-designed questionnaire; | NA |
| Alireza Sadeghi 2019 ^9^ | 13 | cohort study, case-control study | endometrial cancer | OR, RR, HR | FFQ | Q4 vs. Q1 (≥136.1 vs. <109.1 g/d)  Q5 vs. Q1 (≥214 vs. <141 g/d)  Q4 vs. Q1 (≥190 vs. <162 g/d)  Q5 vs. Q1 (≥246 vs. <211 g/d)  Q5 vs. Q1 (≥227 vs. <179 g/d)  Q4 vs. Q1 (≥350 vs. <251.6 g/d) |
| Lai lai Fan 2018 ^36^ | 21 | cohort study, case-control study | prostate cancer | OR | FFQ; Health questionnaire; food diaries | Q4 vs. Q1  Q5 vs. Q1  T3 vs. T1  Dichotomy 1 vs. Dichotomy 2 |
| Sabrina Schlesinger 2017 ^37^ | 14 | cohort study, nested case-control study | breast cancer | RR | FFQ; 24h recall; Dietary history | 59.2 vs 40.6% of energy  >244.1 vs. <185.3 g/d  >305.7 vs. <112.3 g/d  ≥246 vs. <211g/d  343.5 vs. 257.5 g/d  >249 vs. <143 g/d  240 vs. 159 g/d  217.6–303.4 vs. <190.2 g/d  ≥225 vs. <198 g/d  ≥252.7 vs. <181 g/d  ≥278 vs. ≤207 g/d  per 50 g/d |
| Rongjiang Wang 2015 ^38^ | 27 | cohort study, case-control study | prostate cancer | RR | NA | NA |
| Zhongqin Jin 2018 ^44^ | 8 | cohort study, case-control study | inflammatory bowel diseases | OR | NA | NA |

Supplementary table 3. *Continued*

| Individual study | N of studies | Study design | Outcome | Type of effect metric | Methods of exposure assessment | Category and amount of comparison in each primary study |
| --- | --- | --- | --- | --- | --- | --- |
| D. Aune 2012 (a) ^33^ | 14 | cohort study | colorectal cancer | RR | FFQ; 24h recall | 302.3 vs. 242.2 g/day  ≥331.2 vs. <243.9 g/day  ≥281.1 vs. <234.5 g/day  ≥260.1 vs. <131.6 g/day  >162 vs. <114 g/day  ≥246 vs. <211 g/day  ≥275.6 vs. ≤153 g/day  288 vs. 182 g/day  202 vs. 110 g/day  267 vs. 177 g/day  ≥249 vs. <143 g/day  Q 4 vs. Q1  ≥54 vs. <40% of energy  >274 vs. <152 g/day  per 100 g/d |
| Long Zhai 2015 ^41^ | 18 | cohort study, case-control study | prostate cancer | RR | Validated modiﬁed diet history method; FFQ; 24h recall; Healthy questionnaire; | >300 vs. <199.9 g/day  >354 vs. ≤206 g/day  Q4 vs. Q1  >143.23 vs. ≤129.35 g/day  T3 vs. T1 g/day  301.8 vs. 188.5 g/day  343 vs. 234 g/day  287.1 vs. 164.8 g/day  220.8–305.6 vs. 98.2–164.3 g/day  >392 vs. ≤252 g/day  T3 vs. T1  ≥263.8 vs. ≤188.4 g/day  Q4 vs. Q1  ≥350.1 vs. ≤188.6 g/day  214 vs. 333.7 g/day |

Supplementary table 3. *Continued*

| Individual study | N of studies | Study design | Outcome | Type of effect metric | Exposure assessment | Category and amount of comparison in each primary study |
| --- | --- | --- | --- | --- | --- | --- |
| Fei Xuan 2020 ^39^ | 13 | case-control study | esophageal cancer, esophageal adenocarcinoma, esophageal squamous cell carcinoma | OR | HHHQ; FFQ; | NA |
| Xianlei Cai 2019 ^32^ | 44 | cohort study, case-control study | digestive system cancers, Colorectal cancer, Esophageal cancer, gastric cancer, Liver cancer, Pancreatic cancer | RR, OR | SQ-FFQ; FFQ | NA |
| D. Aune 2012 (b) ^34^ | 13 | cohort study | pancreatic cancer risk | RR | FFQ | 285 vs. 203 g/day  ≥151.5 vs. 9.0 units/day  256 vs. 155 g/day  ≥58.7 vs. < 46.7 g/1000 kcal/day  >218.93 vs. ≤162.56 g/day  >177.15 vs. ≤129.98 g/day  >236 vs. < 152 g/day  >330.2 vs. ≤260.7 g/day  202 vs. 110 g/day  >238 vs. ≤178 g/day  per 100 g/day |
| Jian Huang 2017 ^10^ | 17 | cohort study, case-control study | colorectal cancer | RR | NA | NA |
| Geoffrey Livesey 2019 ^11^ | 14 | cohort study | coronary heart disease | RR | FFQ | Per 98 g/d |
| Dale S. Hardy 2020 ^15^ | 40 | cohort study | coronary heart disease, stroke, mortality | HR | FFQ; 24h dietary recall | Q4 vs. Q1  Q5 vs. Q1  T3 vs. T1  continuous |
| Darren C. G Reenwood 2013 ^43^ | 21 | cohort study | type 2 Diabetes | RR | FFQ | per 50g/day |
| Amani Alhazmi 2013 ^12^ | 22 | cohort study | type 2 Diabetes | RR | FFQ | Q4 vs. Q1  Q5 vs. Q1  T3 vs. T1  Decile 10 vs 1 % E  SD increase |

Supplementary table 3. *Continued*

| Individual study | N of studies | Study design | Outcome | Type of effect metric | Exposure assessment | Category and amount of comparison in each primary study |
| --- | --- | --- | --- | --- | --- | --- |
| Yashu Liu 2019 ^13^ | 18 | cohort study, cross-sectional study | metabolic syndrome | OR | 24h dietary recall; FFQ; Diet History survey; 5-day estimated diet records | per 5% energy increment |
| Aimin Wang 2015 ^48^ | 14 | cohort study, case-control study | Parkinson’s disease | RR | NA | 1 SD more  63.6 vs. 46.2 % energy  Q4 vs. Q1  57.6 vs. 36.4–50.5 vs. 27.4 % energy  >239.85 vs. ≤138.38 g/day |
| Xianlei Cai 2015 ^42^ | 7 | cohort study | stroke, hemorrhagic and ischemic stroke mortality | RR | FFQ | NA |
| Fan Wang 2016 ^45^ | 9 | cohort study, case-control study | ulcerative colitis | RR | FFQ | Q5 vs. Q1  Q4 vs. Q1  T3 vs. T1  per 10g increment/day |
| Lirong Zeng 2017 ^46^ | 9 | cohort study, case-control study | Crohn’s disease | RR | FFQ | Q5 vs. Q1  Q4 vs. Q1  T3 vs. T1  per 10g increment/day |
| Sara B Seidelmann 2018 ^8^ | 7 | cohort study | all-cause mortality | HR | NA | NA |
| H. Mozaffari 2020 ^47^ | 5 | cohort study, case-control study | bone fracture | OR, RR, HR | FFQ; 24h dietary recall | per increasing quintile  per 1 standard deviation increment  Calculated (meeting WHO recommendations)  Q5 vs. Q1  Q4 vs. Q1 |

OR, odds ratios (ORs); HR, hazard ratios (HRs); RR, relative risks. NA, not available; FFQ food frequency questionnaire; HHHQ, Health habits and history questionnaire; SQ-FFQ, semi-quantitative food frequency questionnaires; Q: quartile or quintile; T, tertile; h, hour

Supplementary Table 4. References of studies excluded in the umbrella review.

| **Review without meta-analysis (n = 69)** |
| --- |
| 1. Viani K, Albuquerque L, Barr RD Ladas EJ (2020) Nutrition of children with cancer in Brazil: A systematic review. Journal of Global Oncology 6, 242-259.  2. Tosatti JAG, Alves MT Gomes KB (2020) The Role of the Mediterranean Dietary Pattern on Metabolic Control of Patients with Diabetes Mellitus: A Narrative Review. Advances in experimental medicine and biology.  3. Rychter AM, Ratajczak AE, Zawada A, Dobrowolska A Krela-Kazmierczak I (2020) Non-Systematic Review of Diet and Nutritional Risk Factors of Cardiovascular Disease in Obesity. Nutrients 12.  4. Rao M, Gao C, Hou J, Gu J, Law BYK Xu Y (2020) Non-Digestible Carbohydrate and the Risk of Colorectal Neoplasia: A Systematic Review. Nutrition and Cancer-an International Journal.  5. Park MY, Kim J, Chung N, Park HY, Hwang H, Han JS, So JM, Lee CH, Park J Lim K (2020) Dietary factors and eating behaviors affecting diet-induced thermogenesis in obese individuals: A systematic review. Journal of Nutritional Science and Vitaminology 66, 1-9.  6. Litvak J, Parekh N Deierlein A (2020) Prenatal dietary exposures and offspring body size from 6 months to 18 years: A systematic review. Paediatric and Perinatal Epidemiology 34, 171-189.  7. Elma O, Yilmaz ST, Deliens T, Coppieters I, Clarys P, Nijs J Malfliet A (2020) Do Nutritional Factors Interact with Chronic Musculoskeletal Pain? A Systematic Review. Journal of Clinical Medicine 9.  8. van Elten TM, Karsten MDA, van Poppel MNM, Geelen A, Limpens J, Roseboom TJ Gemke R (2019) Diet and physical activity in pregnancy and offspring's cardiovascular health: a systematic review. J Dev Orig Health Dis 10, 286-298.  9. Thornhill K, Charlton K, Probst Y Neale E (2019) Does an increased intake of added sugar affect appetite in overweight or obese adults, when compared with lower intakes? A systematic review of the literature. British Journal of Nutrition 121, 232-240.  10. Song G, Wang Y, Chen X, Pang S, Miao H, Li A Wang W (2019) Influences of ratio of macro-nutrients intake to human and animals: an overview. Food and Agricultural Immunology 30, 190-207.  11. Riccardi G & Costabile G (2019) Carbohydrate quality is key for a healthy and sustainable diet. Nature Reviews Endocrinology 15, 257-258.  12. Kirkpatrick CF, Bolick JP, Kris-Etherton PM, Sikand G, Aspry KE, Soffer DE, Willard KE Maki KC (2019) Review of current evidence and clinical recommendations on the effects of low-carbohydrate and very-low-carbohydrate (including ketogenic) diets for the management of body weight and other cardiometabolic risk factors: A scientific statement from the National Lipid Association Nutrition and Lifestyle Task Force. Journal of Clinical Lipidology 13, 689-711.e681.  13. Jurik R & Stastny P (2019) Role of Nutrition and Exercise Programs in Reducing Blood Pressure: A Systematic Review. Journal of Clinical Medicine 8.  14. Churuangsuk C, Griffiths D, Lean MEJ Combet E (2019) Impacts of carbohydrate-restricted diets on micronutrient intakes and status: A systematic review. Obesity Reviews 20, 1132-1147.  15. Chapman NA, Jacobs RJ Braakhuis AJ (2019) Role of diet and food intake in age-related macular degeneration: a systematic review. Clinical and Experimental Ophthalmology 47, 106-127.  16. Albataineh SR, Badran EF Tayyem RF (2019) Dietary factors and their association with childhood obesity in the Middle East: A systematic review. Nutrition and Health (Bicester) 25, 53-60.  17. Wong MYZ, Man REK, Fenwick EK, Gupta P, Li LJ, van Dam RM, Chong MF Lamoureux EL (2018) Dietary intake and diabetic retinopathy: A systematic review. PLoS ONE 13.  18. Weigl J, Hauner H Hauner D (2018) Can Nutrition Lower the Risk of Recurrence in Breast Cancer? Breast Care 13, 86-91.  19. Turton JL, Raab R Rooney KB (2018) Low-carbohydrate diets for type 1 diabetes mellitus: A systematic review. Plos One 13.  20. Tappy L, Morio B, Azzout-Marniche D, Champ M, Gerber M, Houdart S, Mas E, Rizkalla S, Slama G, Mariotti F et al. (2018) French Recommendations for Sugar Intake in Adults: A Novel Approach Chosen by ANSES. Nutrients 10.  21. Singh R, Salem A, Nanavati J Mullin GE (2018) The Role of Diet in the Treatment of Irritable Bowel Syndrome: A Systematic Review. Gastroenterology Clinics of North America 47, 1-22.  22. Makarem N, Bandera EV, Nicholson JM Parekh N (2018) Consumption of Sugars, Sugary Foods, and Sugary Beverages in Relation to Cancer Risk: A Systematic Review of Longitudinal Studies. In Annual Review of Nutrition, Vol 38, vol. 38, pp. 17-39 [PJ Stover and R Balling, editors].  23. Duncanson KR, Talley NJ, Walker MM Burrows TL (2018) Food and functional dyspepsia: a systematic review. Journal of Human Nutrition and Dietetics 31, 390-407.  24. Dow C, Mancini F, Rajaobelina K, Boutron-Ruault MC, Balkau B, Bonnet F Fagherazzi G (2018) Diet and risk of diabetic retinopathy: a systematic review. European Journal of Epidemiology 33, 141-156.  25. Churuangsuk C, Kherouf M, Combet E Lean M (2018) Low-carbohydrate diets for overweight and obesity: a systematic review of the systematic reviews. Obesity Reviews 19, 1700-1718.  26. Boyle NB, Lawton CL Dye L (2018) The Effects of Carbohydrates, in Isolation and Combined with Caffeine, on Cognitive Performance and MoodCurrent Evidence and Future Directions. Nutrients 10.  27. Zalewski BM, Patro B, Veldhorst M, Kouwenhoven S, Crespo Escobar P, Calvo Lerma J, Koletzko B, van Goudoever JB Szajewska H (2017) Nutrition of infants and young children (one to three years) and its effect on later health: A systematic review of current recommendations (EarlyNutrition project). Critical reviews in food science and nutrition 57, 489-500.  28. Wong M, Man R, Gupta P, Fenwick E, Li LJ Lamoureux E (2017) A systematic review of the associations between dietary intake and diabetic retinopathy. Acta Ophthalmologica 95.  29. Schürmann S, Kersting M Alexy U (2017) Vegetarian diets in children: a systematic review. Eur J Nutr 56, 1797-1817.  30. Patkova A, Joskova V, Havel E, Kovarik M, Kucharova M, Zadak Z Hronek M (2017) Energy, Protein, Carbohydrate, and Lipid Intakes and Their Effects on Morbidity and Mortality in Critically Ill Adult Patients: A Systematic Review. Advances in Nutrition 8, 624-634.  31. McCormick NM & Logomarsino JV (2017) The specific carbohydrate diet in the treatment of Crohn's disease: A systematic review. Journal of Gastroenterology and Hepatology Research 6, 2392-2399.  32. Khong TK, Selvanayagam VS, Sidhu SK Yusof A (2017) Role of carbohydrate in central fatigue: a systematic review. Scandinavian Journal of Medicine & Science in Sports 27, 376-384.  33. Keikha M, Bahreynian M, Saleki M Kelishadi R (2017) Macro- and Micronutrients of Human Milk Composition: Are They Related to Maternal Diet? A Comprehensive Systematic Review. Breastfeeding Medicine 12, 517-527.  34. Doaei S, Kalantari N, Mohammadi NK, Tabesh GA Gholamalizadeh M (2017) Macronutrients and the FTO gene expression in hypothalamus; a systematic review of experimental studies. Indian heart journal 69, 277-281.  35. Whelan ME, Wright OR Hickman IJ (2016) A Review of the Effect of Dietary Composition on Fasting Substrate Oxidation in Healthy and Overweight Subjects. Critical reviews in food science and nutrition 56, 146-151.  36. Veena SR, Gale CR, Krishnaveni GV, Kehoe SH, Srinivasan K Fall CHD (2016) Association between maternal nutritional status in pregnancy and offspring cognitive function during childhood and adolescence; a systematic review. Bmc Pregnancy and Childbirth 16.  37. Tielemans MJ, Garcia AH, Peralta Santos A, Bramer WM, Luksa N, Luvizotto MJ, Moreira E, Topi G, de Jonge EA, Visser TL et al. (2016) Macronutrient composition and gestational weight gain: a systematic review. Am J Clin Nutr 103, 83-99.  38. Thom NJ, Early AR, Hunt BE, Harris RA Herring MP (2016) Eating and arterial endothelial function: a meta-analysis of the acute effects of meal consumption on flow-mediated dilation. Obesity Reviews 17, 1080-1090.  39. Stanhope KL (2016) Sugar consumption, metabolic disease and obesity: The state of the controversy. Critical Reviews in Clinical Laboratory Sciences 53, 52-67.  40. Schoenaker DA, Mishra GD, Callaway LK Soedamah-Muthu SS (2016) The Role of Energy, Nutrients, Foods, and Dietary Patterns in the Development of Gestational Diabetes Mellitus: A Systematic Review of Observational Studies. Diabetes Care 39, 16-23.  41. Sartorius B, Sartorius K, Aldous C, Madiba TE, Stefan C Noakes T (2016) Carbohydrate intake, obesity, metabolic syndrome and cancer risk? A two-part systematic review and meta-analysis protocol to estimate attributability. Bmj Open 6.  42. Rocha NP, Milagres LC, De Novaes JF Do Carmo Castro Franceschini S (2016) Association between food and nutrition insecurity with cardiometabolic risk factors in childhood and adolescence: A systematic review. Revista Paulista de Pediatria 34, 225-233.  43. Kaartinen NE, Knekt P, Kanerva N, Valsta LM, Eriksson JG, Rissanen H, Jaaskelainen T Mannisto S (2016) Dietary carbohydrate quantity and quality in relation to obesity: A pooled analysis of three Finnish population-based studies. Scandinavian Journal of Public Health 44, 385-393.  44. Frary JMC, Bjerre KP, Glintborg D Ravn P (2016) The effect of dietary carbohydrates in women with polycystic ovary syndrome: a systematic review. Minerva Endocrinologica 41, 57-69.  45. Bravi F, Wiens F, Decarli A, Dal Pont A, Agostoni C Ferraroni M (2016) Impact of maternal nutrition on breast-milk composition: A systematic review. American Journal of Clinical Nutrition 104, 646-662.  46. Mourouti N, Kontogianni MD, Papavagelis C Panagiotakos DB (2015) Diet and breast cancer: a systematic review. International Journal of Food Sciences and Nutrition 66, 1-42.  47. Louie JC & Tapsell LC (2015) Association between intake of total vs added sugar on diet quality: a systematic review. Nutr Rev 73, 837-857.  48. de Waure C, Quaranta G, Gualano MR, Cadeddu C, Jovic-Vranes A, Djikanovic B, La Torre G Ricciardi W (2015) Systematic review of studies investigating the association between dietary habits and cutaneous malignant melanoma. Public Health 129, 1099-1113.  49. Azeem S, Gillani SW, Siddiqui A, Jandrajupalli SB, Poh V Syed Sulaiman SA (2015) Diet and Colorectal Cancer Risk in Asia--a Systematic Review. Asian Pacific journal of cancer prevention : APJCP 16, 5389-5396.  50. Yanai H, Katsuyama H, Hamasaki H, Abe S, Tada N Sako A (2014) Effects of Carbohydrate and Dietary Fiber Intake, Glycemic Index and Glycemic Load on HDL Metabolism in Asian Populations. Journal of clinical medicine research 6, 321-326.  51. Moynihan PJ & Kelly SA (2014) Effect on caries of restricting sugars intake: systematic review to inform WHO guidelines. J Dent Res 93, 8-18.  52. Freeman R (2014) Moderate evidence support a relationship between sugar intake and dental caries. Evidence-based dentistry 15, 98-99.  53. Morenga LT (2013) Epidemiology of sugar in the development of disease and population health. Obesity Research and Clinical Practice 7, e29.  54. Masko EM, Allott EH Freedland SJ (2013) The Relationship Between Nutrition and Prostate Cancer: Is More Always Better? European Urology 63, 810-820.  55. Wheeler ML, Dunbar SA, Jaacks LM, Karmally W, Mayer-Davis EJ, Wylie-Rosett J Yancy WS, Jr. (2012) Macronutrients, Food Groups, and Eating Patterns in the Management of Diabetes A systematic review of the literature, 2010. Diabetes Care 35, 434-445.  56. Fogelholm M, Anderssen S, Gunnarsdottir I Lahti-Koski M (2012) Dietary macronutrients and food consumption as determinants of long-term weight change in adult populations: a systematic literature review. Food & Nutrition Research 56.  57. Hou JK, Abraham B El-Serag H (2011) Dietary Intake and Risk of Developing Inflammatory Bowel Disease: A Systematic Review of the Literature. American Journal of Gastroenterology 106, 563-573.  58. Aller EE, Abete I, Astrup A, Martinez JA van Baak MA (2011) Starches, sugars and obesity. Nutrients 3, 341-369.  59. Ahmadi-Abhari S & Chowdhury R (2011) DIETARY INTAKE OF CARBOHYDRATES AND RISK OF TYPE 2 DIABETES: A SYSTEMATIC REVIEW AND META-ANALYSIS. Journal of Epidemiology and Community Health 65, A220-A221.  60. Lee CT, Gayton EL, Beulens JW, Flanagan DW Adler AI (2010) Micronutrients and diabetic retinopathy a systematic review. Ophthalmology 117, 71-78.  61. Mente A, de Koning L, Shannon HS Anand SS (2009) A Systematic Review of the Evidence Supporting a Causal Link Between Dietary Factors and Coronary Heart Disease. Archives of Internal Medicine 169, 659-669.  62. Hoyland A, Lawton CL Dye L (2008) Acute effects of macronutrient manipulations on cognitive test performance in healthy young adults: A systematic research review. Neuroscience and Biobehavioral Reviews 32, 72-85.  63. Myers VH & Champagne CM (2007) Nutritional effects on blood pressure. Current Opinion in Lipidology 18, 20-24.  64. Key TJ & Spencer EA (2007) Carbohydrates and cancer: an overview of the epidemiological evidence. European Journal of Clinical Nutrition 61, S112-S121.  65. Gibson SA (2007) Dietary sugars intake and micronutrient adequacy: a systematic review of the evidence. Nutrition Research Reviews 20, 121-131.  66. Benamouzig R & Airinei G (2007) Diet and reflux. Journal of Clinical Gastroenterology 41, S64-S71.  67. Saris WHM (2006) Macronutrient intake balance and the problem of obesity - Old recipes and some new spices. Aktuelle Ernahrungsmedizin 31, S49-S54.  68. Krieger JW, Sitren HS, Danieis MJ Langkamp-Henken B (2006) Meta-regression analysis of low carbohydrate variable protein energy-restricted diet studies on weight loss and body composition in humans. Faseb Journal 20, A582-A582.  69. Murakami K, Okubo H Sasaki S (2005) Effect of dietary factors on incidence of type 2 diabetes: a systematic review of cohort studies. J Nutr Sci Vitaminol (Tokyo) 51, 292-310. |
| **Study specific data missing (n = 5)** |
| 1. Kim J, Hoang T, Bu SY, Kim J-M, Choi J-H, Park E, Park E, 이승민, 민지연, 이인석 et al. (2020) Associations of Dietary Intake with Cardiovascular Disease, Blood Pressure, and Lipid Profile in the Korean Population: a Systematic Review and Meta-Analysis. Journal of Lipid and Atherosclerosis 9, 205-229.  2. Sartorius K, Sartorius B, Madiba TE Stefan C (2018) Does high-carbohydrate intake lead to increased risk of obesity? A systematic review and meta-analysis. Bmj Open 8.  3. Wu H, Zhang H, Li P, Gao T, Lin J, Yang J, Wu Y Ye J (2014) Association Between Dietary Carbohydrate Intake and Dietary Glycemic Index and Risk of Age-Related Cataract: A Meta-Analysis. Investigative Ophthalmology & Visual Science 55, 3660-3668.  4. Chu KT, Song Y Zhou JH (2014) No effect of energy intake overall on risk of endometrial cancers: a meta-analysis. Asian Pacific journal of cancer prevention : APJCP 15, 10293-10298.  5. Sharp WG, Berry RC, McCracken C, Nuhu NN, Marvel E, Saulnier CA, Klin A, Jones W Jaquess DL (2013) Feeding problems and nutrient intake in children with autism spectrum disorders: A meta-analysis and comprehensive review of the literature. Journal of Autism and Developmental Disorders 43, 2159-2173. |
| **Publications not in English (n = 8)** |
| 1. Jing S, Li H Ruopeng A (2019) Food environment and its relation to diet behavior and obesity in China. Chinese Journal of Endemiology 40, 1296-1303.  2. Krejci H, Vyjidak J Kohutiar M (2018) Low-carbohydrate diet in diabetes mellitus treatment. Vnitrni lekarstvi 64, 742-752.  3. Valenzuela Mencia J, Fernandez Castillo R, Martos Cabrera MB, Luis Gomez-Urquiza J, Albendin Garcia L Canadas de la Fuente GA (2017) Diets low in carbohydrates for type 2 diabetics. Systematic review. Nutricion Hospitalaria 34, 224-234.  4. Shao D, Wang H, Min J Wang F (2016) A Systematic Review of Dietary Factors and Gastrointestinal Tract Cancer. Genomics and Applied Biology 35, 1101-1107.  5. Delgado-Noguera M, Gallego JM Maya JD (2016) Effectiveness of carbohydrate counting for metabolic control of children with type 1 diabetes mellitus: Systematic review. Revista Argentina de Endocrinologia y Metabolismo 53, 142-148.  6. Fontan JdS & Amadio MB (2015) USE OF CARBOHYDRATE BEFORE PHYSICAL ACTIVITY AS ERGOGENIC AID: A SYSTEMATIC REVIEW. Revista Brasileira De Medicina Do Esporte 21, 153-157.  7. Luna Lopez V, Lopez Medina JA, Vazquez Gutierrez M Fernandez Soto ML (2014) CARBOHYDRATE: CURRENT ROLE IN DIABETES MELLITUS AND METABOLIC DISEASE. Nutricion Hospitalaria 30, 1020-1031.  8. Morozov SV (2013) The role of nutritional factors in pathogenesis and treatment of functional dyspepsia. Klinicheskie perspektivy gastroenterologii, gepatologii, 56-64. |
| **Meeting abstract (n = 15)** |
| 1. Hasan I & Deaningtyas P (2020) Impact of low-carbohydrate diet compared to low-fat diet on hepatic fat content in patients with non-alcoholic fatty liver disease. Hepatology International 14, S372.  2. Yanagisawa H, Toko H, Harada M, Guo J, Bujo S, Ishizuka M Komuro I (2019) The effects of low-carbohydrate high-fat diet on vascular remodeling. Circulation Research 125.  3. Sacks F (2018) Fatty acids and carbohydrates in healthful dietary patterns to prevent cardiovascular disease. Nutrition and Metabolism 15.  4. Churuangsuk C, Kherouf M, Lean MEJ Combet E (2018) Weight reduction following low-carbohydrate diets compared to low-fat diets: A systematic review and quality assessment of systematic reviews with meta-analyses. Obesity Facts 11, 306.  5. McArdle PD, Gill P Greenfield S (2017) Quantity of Carbohydrate in Type 2 Diabetes: A Systematic Review. Diabetes 66, A206-A206.  6. Harlow K, Yu E, Joshi S, Goyal N, Newton K Schwimmer J (2017) Association of dietary carbohydrates and sugars with nonalcoholic fatty liver disease: A systematic review. Journal of Pediatric Gastroenterology and Nutrition 65, S169-S170.  7. Li J, Wright CS Campbell WW (2016) Carbohydrate Intake Affects Weight Loss Related Improvements in Glycemic Control: Results from a Systematic Review and Regression Analysis. Faseb Journal 30.  8. Chuah KA, Yeak ZW, Balasubramanian G, Se CH, Ng SH, Sahathevan S, Chinna K, Sundram K Karupaiah T (2016) Carbohydrates but not Fats Trigger Coronary Heart Disease Risk: Findings from the Malaysia Lipid Study (MLS). Faseb Journal 30.  9. Van Wyk HJ, Davies JS Davis RE (2015) A critical review of meta-analyses of low carbohydrate diets in subjects with Type 2 diabetes. Diabetic Medicine 32, 52-52.  10. Boyle P, Boniol M, Koechlin A, Bota M, Pizot C Autier P (2015) Sugar and fructose consumption and the risk of cancer. Journal of Clinical Oncology 33.  11. Mullie P, Autier P Boyle P (2013) Overview of meta- and pooled analyses of nutrition and breast, colorectal and prostate cancer risk. European Journal of Cancer 49, S317-S318.  12. Boaz M, Jakubowicz D Wainstein J (2013) Macronutrient Composition in Weight Loss Diets: A Meta-Analysis. Diabetes 62, A401-A401.  13. Aune D, Navarro Rosenblatt D, Chan DSM, Vieira AR, Vieira R Norat T (2012) Dietary carbohydrates, glycemic index, glycemic load and endometrial cancer risk: A systematic review and meta-analysis of prospective studies. Proceedings of the Nutrition Society 71.  14. Chia JSL, Yang J, Khor MJ Li XY (2011) A systematic review on the impact of carbohydrate counting on glycaemic control in type 1 diabetes. Proceedings of Singapore Healthcare 20, 61.  15. Kodama S, Saito K, Yachi Y, Ibe Y, Asumi M, Maki M, Suzuki E, Shimano H, Yamada N Sone H (2008) Effect of different dietary carbohydrate/fat ratios on glucose metabolism in type 2 diabetic patients: A meta-analysis. Diabetes 57, A470-A470. |
| **Meta-analysis of intervention study (n = 32)** |
| 1. Schwingshackl L, Neuenschwander M, Hoffmann G, Buyken AE Schlesinger S (2020) Dietary sugars and cardiometabolic risk factors: a network meta-analysis on isocaloric substitution interventions. American Journal of Clinical Nutrition 111, 187-196.  2. Porchia LM, Celeste Hernandez-Garcia S, Elba Gonzalez-Mejia M Lopez-Bayghen E (2020) Diets with lower carbohydrate concentrations improve insulin sensitivity in women with polycystic ovary syndrome: A meta-analysis. European Journal of Obstetrics & Gynecology and Reproductive Biology 248, 110-117.  3. Kloby Nielsen LL, Tandrup Lambert MN Jeppesen PB (2020) The Effect of Ingesting Carbohydrate and Proteins on Athletic Performance: A Systematic Review and Meta-Analysis of Randomized Controlled Trials. Nutrients 12.  4. Kdekian A, Alssema M, Van Der Beek EM, Greyling A, Vermeer MA, Mela DJ Trautwein EA (2020) Impact of isocaloric exchanges of carbohydrate for fat on postprandial glucose, insulin, triglycerides, and free fatty acid responses—a systematic review and meta-analysis. European Journal of Clinical Nutrition 74.  5. Fechner E, Smeets ETHC, Schrauwen P Mensink RP (2020) The effects of different degrees of carbohydrate restriction and carbohydrate replacement on cardiometabolic risk markers in humans—a systematic review and meta-analysis. Nutrients 12.  6. Bueno NB, dos Santos Silva MM de Melo ISV (2020) Low-carbohydrate diets and intrahepatic lipid content in individuals with non-alcoholic fatty liver disease: Evidence from a meta-analysis of randomized trials. Clinical Nutrition 39, 310-311.  7. Zhang X, Zheng Y, Guo Y Lai Z (2019) The Effect of Low Carbohydrate Diet on Polycystic Ovary Syndrome: A Meta-Analysis of Randomized Controlled Trials. International Journal of Endocrinology 2019.  8. Zafar MI, Mills KE, Zheng J, Regmi A, Hu SQ, Gou L Chen LL (2019) Low-glycemic index diets as an intervention for diabetes: a systematic review and meta-analysis. Am J Clin Nutr 110, 891-902.  9. Schwingshackl L, Chaimani A, Schwedhelm C, Toledo E, Pünsch M, Hoffmann G Boeing H (2019) Comparative effects of different dietary approaches on blood pressure in hypertensive and pre-hypertensive patients: A systematic review and network meta-analysis. Critical reviews in food science and nutrition 59, 2674-2687.  10. Rao M, Gao C, Xu L, Jiang L, Zhu J, Chen G, Law BYK Xu Y (2019) Effect of Inulin-Type Carbohydrates on Insulin Resistance in Patients with Type 2 Diabetes and Obesity: A Systematic Review and Meta-Analysis. Journal of Diabetes Research 2019.  11. Papamichou D, Panagiotakos DB Itsiopoulos C (2019) Dietary patterns and management of type 2 diabetes: A systematic review of randomised clinical trials. Nutrition Metabolism and Cardiovascular Diseases 29, 531-543.  12. Pan B, Wu Y, Yang Q, Ge L, Gao C, Xun Y, Tian J Ding G (2019) The impact of major dietary patterns on glycemic control, cardiovascular risk factors, and weight loss in patients with type 2 diabetes: A network meta-analysis. Journal of evidence-based medicine 12, 29-39.  13. McArdle PD, Greenfield SM, Rilstone SK, Narendran P, Haque MS Gill PS (2019) Carbohydrate restriction for glycaemic control in Type 2 diabetes: a systematic review and meta-analysis. Diabetic Medicine 36, 335-348.  14. Mantantzis K, Schlaghecken F, Sünram-Lea SI Maylor EA (2019) Sugar rush or sugar crash? A meta-analysis of carbohydrate effects on mood. Neuroscience and Biobehavioral Reviews 101, 45-67.  15. Korsmo-Haugen H-K, Brurberg KG, Mann J Aas A-M (2019) Carbohydrate quantity in the dietary management of type 2 diabetes: A systematic review and meta-analysis. Diabetes Obesity & Metabolism 21, 15-27.  16. Jovanovski E, de Castro Ruiz Marques A, Li D, Ho HVT, Blanco Mejia S, Sievenpiper JL, Zurbau A, Komishon A, Duvnjak L, Bazotte RB et al. (2019) Effect of high-carbohydrate or high-monounsaturated fatty acid diets on blood pressure: a systematic review and meta-analysis of randomized controlled trials. Nutr Rev 77, 19-31.  17. Gjuladin-Hellon T, Davies IG, Penson P Amiri Baghbadorani R (2019) Effects of carbohydrate-restricted diets on low-density lipoprotein cholesterol levels in overweight and obese adults: a systematic review and meta-analysis. Nutr Rev 77, 161-180.  18. Brietzke C, Franco-Alvarenga PE, Coelho-Júnior HJ, Silveira R, Asano RY Pires FO (2019) Effects of Carbohydrate Mouth Rinse on Cycling Time Trial Performance: A Systematic Review and Meta-Analysis. Sports medicine (Auckland, NZ) 49, 57-66.  19. Vaz EC, Martiniano Porfirio GJ, de Carvalho Nunes HR Nunes-Nogueira VdS (2018) Effectiveness and safety of carbohydrate counting in the management of adult patients with type 1 diabetes mellitus: a systematic review and meta-analysis. Archives of Endocrinology Metabolism 62, 337-345.  20. van Zuuren EJ, Fedorowicz Z, Kuijpers T Pijl H (2018) Effects of low-carbohydrate- compared with low-fat-diet interventions on metabolic control in people with type 2 diabetes: a systematic review including GRADE assessments. American Journal of Clinical Nutrition 108, 300-331.  21. McCartney D, Desbrow B Irwin C (2018) Post-exercise Ingestion of Carbohydrate, Protein and Water: A Systematic Review and Meta-analysis for Effects on Subsequent Athletic Performance. Sports Medicine 48, 379-408.  22. Huntriss R, Campbell M Bedwell C (2018) The interpretation and effect of a low-carbohydrate diet in the management of type 2 diabetes: a systematic review and meta-analysis of randomised controlled trials. European Journal of Clinical Nutrition 72.  23. Amissah EA, Brown J Harding JE (2018) Carbohydrate supplementation of human milk to promote growth in preterm infants. Cochrane Database of Systematic Reviews.  24. Ahmed S, Singh D, Khattab S, Babineau J Kumbhare D (2018) The effects of Diet on the Proportion of intramuscular Fat in Human Muscle: A Systematic Review and Meta-analysis. Frontiers in Nutrition 5.  25. von Bibra H, Ströhle A, St. John Sutton M Worm N (2017) Dietary therapy in heart failure with preserved ejection fraction and/or left ventricular diastolic dysfunction in patients with metabolic syndrome. International Journal of Cardiology 234, 7-15.  26. Fu S, Li L, Deng S, Zan L Liu Z (2016) Effectiveness of advanced carbohydrate counting in type 1 diabetes mellitus: a systematic review and meta-analysis. Scientific Reports 6.  27. Bell KJ, Barclay AW, Petocz P, Colagiuri S Brand-Miller JC (2014) Efficacy of carbohydrate counting in type 1 diabetes: a systematic review and meta-analysis. Lancet Diabetes & Endocrinology 2, 133-140.  28. Schwingshackl L & Hoffmann G (2013) Low-carbohydrate diets impair flow-mediated dilatation: evidence from a systematic review and meta-analysis. British Journal of Nutrition 110, 969-970.  29. Bell K, Barclay AW, Petocz P, Colagiuri S Brand-Miller JC (2013) The efficacy of carbohydrate countings in type 1 diabetes: A systematic review and meta-analysis. Diabetes 62, A97.  30. Stearns RL, Emmanuel H, Volek JS Casa DJ (2010) EFFECTS OF INGESTING PROTEIN IN COMBINATION WITH CARBOHYDRATE DURING EXERCISE ON ENDURANCE PERFORMANCE: A SYSTEMATIC REVIEW WITH META-ANALYSIS. Journal of Strength and Conditioning Research 24, 2192-2202.  31. Kodama S, Saito K, Tanaka S, Maki M, Yachi Y, Sato M, Sugawara A, Totsuka K, Shimano H, Ohashi Y et al. (2009) Influence of Fat and Carbohydrate Proportions on the Metabolic Profile in Patients With Type 2 Diabetes: A Meta-Analysis. Diabetes Care 32, 959-965.  32. Kirk JK, Graves DE, Craven TE, Lipkin EW, Austin M Margolis KL (2008) Restricted-carbohydrate diets in patients with type 2 diabetes: A meta-analysis. Journal of the American Dietetic Association 108, 91-100. |
| **Outcome with genetic expression (n = 2)** |
| 1. Doaei S, Kalantari N, Mohammadi NK, Tabesh GA Gholamalizadeh M (2017) Macronutrients and the FTO gene expression in hypothalamus; a systematic review of experimental studies. Indian heart journal 69, 277-281.  2. Livingstone KM, Celis-Morales C, Lara J, Ashor AW, Lovegrove JA, Martinez JA, Saris WH, Gibney M, Manios Y, Traczyk I et al. (2015) Associations between FTO genotype and total energy and macronutrient intake in adults: a systematic review and meta-analysis. Obesity Reviews 16, 666-678. |
| **Exposure without dietary carbohydrate intake (n = 32)** |
| 1. Ebrahimpour-Koujan S, Saneei P, Larijani B Esmaillzadeh A (2020) Consumption of sugar sweetened beverages and dietary fructose in relation to risk of gout and hyperuricemia: a systematic review and meta-analysis. Crit Rev Food Sci Nutr 60, 1-10.  2. Dong T, Guo M, Zhang P, Sun G Chen B (2020) The effects of low-carbohydrate diets on cardiovascular risk factors: A meta-analysis. Plos One 15.  3. Churuangsuk C, Lean MEJ Combet E (2020) Low and reduced carbohydrate diets: challenges and opportunities for type 2 diabetes management and prevention. The Proceedings of the Nutrition Society, 1-16.  4. Turton J, Brinkworth GD, Field R, Parker H Rooney K (2019) An evidence-based approach to developing low-carbohydrate diets for type 2 diabetes management: A systematic review of interventions and methods. Diabetes Obesity & Metabolism 21, 2513-2525.  5. Reynolds A, Mann J, Cummings J, Winter N, Mete E Te Morenga L (2019) Carbohydrate quality and human health: a series of systematic reviews and meta-analyses. Lancet 393, 434-445.  6. Kwok CS, Gulati M, Michos ED, Potts J, Wu P, Watson L, Loke YK, Mallen C Mamas MA (2019) Dietary components and risk of cardiovascular disease and all-cause mortality: a review of evidence from meta-analyses. European Journal of Preventive Cardiology 26, 1415-1429.  7. Khan TA, Tayyiba M, Agarwal A, Mejia SB, de Souza RJ, Wolever TMS, Leiter LA, Kendall CWC, Jenkins DJA Sievenpiper JL (2019) Relation of Total Sugars, Sucrose, Fructose, and Added Sugars With the Risk of Cardiovascular Disease: A Systematic Review and Dose-Response Meta-analysis of Prospective Cohort Studies. Mayo Clinic Proceedings 94, 2399-2414.  8. Hill E, Goodwill AM, Gorelik A Szoeke C (2019) Diet and biomarkers of Alzheimer's disease: a systematic review and meta-analysis. Neurobiology of Aging 76, 45-52.  9. do Prado SBR, Castro-Alves VC, Ferreira GF Fabi JP (2019) Ingestion of Non-digestible Carbohydrates From Plant-Source Foods and Decreased Risk of Colorectal Cancer: A Review on the Biological Effects and the Mechanisms of Action. Front Nutr 6, 72.  10. Amezketa Garcia L & Martinez-Pineda M (2019) Are High Carbohydrate Low Fat (HCLF) diets fine strategies to approach obesity and overweight? Annals of Nutrition and Metabolism 75, 25-25.  11. Ahn J, Jun DW, Lee HY Moon JH (2019) Critical appraisal for low-carbohydrate diet in nonalcoholic fatty liver disease: Review and meta-analyses. Clinical Nutrition 38, 2023-2030.  12. Tyasnugroho Suyoto PS (2018) Effect of low-carbohydrate diet on markers of renal function in patients with type 2 diabetes: A meta-analysis. Diabetes-Metabolism Research and Reviews 34.  13. Mazidi M, Katsiki N, Mikhailidis DP Banach M (2018) Low-carbohydrate diets and all-cause and cause-specific mortality: A population-based cohort study and pooling prospective studies. European Heart Journal 39, 1112-1113.  14. Lopez-Jaramillo P, Otero J, Anthony Camacho P, Baldeon M Fornasini M (2018) Reevaluating nutrition as a risk factor for cardio-metabolic diseases. Colombia Medica 49, 175-181.  15. Wong SHS, Sun FH, Chen YJ, Li C, Zhang YJ Huang WY (2017) Effect of pre-exercise carbohydrate diets with high vs low glycemic index on exercise performance: A meta-analysis. Nutrition Reviews 75, 327-338.  16. Ruanpeng D, Thongprayoon C, Cheungpasitporn W Harindhanavudhi T (2017) Sugar and artificially sweetened beverages linked to obesity: a systematic review and meta-analysis. QJM : monthly journal of the Association of Physicians 110, 513-520.  17. Namazi N, Larijani B Azadbakht L (2017) Low-Carbohydrate-Diet Score and its Association with the Risk of Diabetes: A Systematic Review and Meta-Analysis of Cohort Studies. Hormone and Metabolic Research 49, 565-571.  18. Ciardulli A, Saccone G, Anastasio H Berghella V (2017) Less-Restrictive Food Intake During Labor in Low-Risk Singleton Pregnancies A Systematic Review and Meta-analysis. Obstetrics and Gynecology 129, 473-480.  19. Carlson JN, Schunder-Tatzber S, Neilson CJ Hood N (2017) Dietary sugars versus glucose tablets for first-aid treatment of symptomatic hypoglycaemia in awake patients with diabetes: a systematic review and meta-analysis. Emergency medicine journal : EMJ 34, 100-106.  20. Khan TA, Blanco-Mejia S, de Souza R, Kendall CWC Sievenpiper JL (2016) Relation of Total Sugars and Fructose-Containing Sugars with Risk of Cardiovascular Disease: A Systematic Review and Meta-Analysis of Prospective Cohort Studies. Faseb Journal 30.  21. Sievenpiper JL (2015) Sugars in obesity and diabetes: Results of systematic reviews and meta-analyses. Annals of Nutrition and Metabolism 67, 16-16.  22. Cheungpasitporn W, Thongprayoon C, Edmonds PJ, Srivali N, Ungprasert P, Kittanamongkolchai W Erickson SB (2015) Sugar and artificially sweetened soda consumption linked to hypertension: A systematic review and meta-analysis. Clinical and Experimental Hypertension 37, 587-593.  23. Zhou J & Xu H (2014) LOW CARBOHYDRATE AND HIGH PROTEIN DIETS AND ALL-CAUSE, CANCER AND CARDIOVASCULAR DISEASES MORTALITIES: A SYSTEMATIC REVIEW AND META-ANALYSIS FROM 7 COHORT STUDIES. Acta Endocrinologica-Bucharest 10, 259-266.  24. Schmidt S, Schelde B Nørgaard K (2014) Effects of advanced carbohydrate counting in patients with Type 1 diabetes: A systematic review. Diabetic Medicine 31, 886-896.  25. Naude CE, Schoonees A, Senekal M, Young T, Garner P Volmink J (2014) Low Carbohydrate versus Isoenergetic Balanced Diets for Reducing Weight and Cardiovascular Risk: A Systematic Review and Meta-Analysis. Plos One 9.  26. Clifton PM, Condo D Keogh JB (2014) Long term weight maintenance after advice to consume low carbohydrate, higher protein diets--a systematic review and meta analysis. Nutrition, metabolism, and cardiovascular diseases : NMCD 24, 224-235.  27. Noto H, Goto A, Tsujimoto T Noda M (2013) Low-Carbohydrate Diets and All-Cause Mortality: A Systematic Review and Meta-Analysis of Observational Studies. Plos One 8.  28. Goletzke J, Buyken AE, Joslowski G, Felbick A, Cheng G, Herder C Brand-Miller JC (2013) The role of carbohydrate quality in chronic low-grade inflammation-a systematic review on observational and intervention studies. Obesity Facts 6, 222.  29. Galeone C, Augustin LSA, Filomeno M, Malerba S, Zucchetto A, Pelucchi C, Montella M, Talamini R, Franceschi S La Vecchia C (2013) Dietary glycemic index, glycemic load, and the risk of endometrial cancer: a case-control study and meta-analysis. European Journal of Cancer Prevention 22, 38-45.  30. Morenga LT, Mallard S Mann J (2012) Dietary sugars and body weight: Systematic review and meta-analyses of randomised controlled trials and cohort studies. BMJ (Online) 345.  31. Galeone C, Pelucchi C La Vecchia C (2012) Added sugar, glycemic index and load in colon cancer risk. Current Opinion in Clinical Nutrition and Metabolic Care 15, 368-373.  32. Anderson JW, Randles KM, Kendall CW Jenkins DJ (2004) Carbohydrate and fiber recommendations for individuals with diabetes: a quantitative assessment and meta-analysis of the evidence. J Am Coll Nutr 23, 5-17. |

Supplementary table 5. The characteristics and quantitative synthesis of all eligible meta-analyses reporting dietary carbohydrate intake relation to multiple outcomes.

| Outcomes | Individual study | No. of primary studies | No. of  cases/ participants | Comparison | Summary relative risk (95% CI) | | Random  *P* value† | Fixed  *P* value‡ |
| --- | --- | --- | --- | --- | --- | --- | --- | --- |
|  |  |  |  |  | Random effects | Fixed effects |  |  |
| Mortality |  |  |  |  |  |  |  |  |
| All-cause mortality | Sara B Seidelmann, 2018 | 5 | 30942/ 287644 | low vs. moderate | 1.19 (1.09-1.30) | 1.14 (1.09-1.20) | 1.456×10^-4^ | 1.785×10^-8^ |
| Stroke and specific-cause mortality* | Xianlei Cai, 2015 | 6 | 1831/ 170348 | high vs. low, dose-response | 1.12 (0.92-1.36) | 1.12 (0.93-1.35) | 0.258 | 0.236 |
| All-cause and specific-cause mortality§ | Dale S. Hardy, 2020 | 5 | 4191/ 110411 | high vs. low | 1.04 (0.91-1.19) | 1.04 (0.91-1.19) | 0.561 | 0.561 |
| Cancer |  |  |  |  |  |  |  |  |
| Breast cancer | Sabrina Schlesinger, 2017 | 11 | 30201/ 885890 | dose-response | 1.00 (0.96-1.05) | 1.00 (0.98-1.02) | 0.955 | 0.953 |
| Colorectal cancer | D. Aune, 2012 (a) | 11 | 9799/ 806647 | high vs. low | 0.93 (0.84-1.04) | 0.92 (0.85-1.00) | 0.223 | 0.038 |
|  | D. Aune, 2012 (a) | 9 | 9246/ 783980 | dose-response | 0.95 (0.85-1.07) | 0.93 (0.87-1.00) | 0.403 | 0.041 |
|  | Xianlei Cai, 2019 | 13 | 10211/ 697179 | high vs. low | 0.99 (0.87-1.12) | 0.96 (0.88-1.04) | 0.852 | 0.323 |
|  | Jian Huang, 2017 | 16 | 11400/ 843184 | high vs. low | 1.08 (0.93-1.26) | 1.01 (0.93-1.10) | 0.308 | 0.791 |
| Digestive system cancers | Xianlei Cai, 2019 | 20 | 11594/ 2666588 | high vs. low | 1.01 (0.93-1.10) | 1.00 (0.93-1.07) | 0.784 | 0.972 |
|  | Xianlei Cai, 2019 | 14 | 5455/ 11823 | high vs. low | 0.76 (0.58-1.01) | 0.84 (0.75-0.93) | 0.056 | 0.001 |
| Endometrial cancer | Alireza Sadeghi, 2019 | 6 | 3998/ 490255 | high vs. low | 1.08 (0.87-1.33) | 1.09 (0.98-1.22) | 0.486 | 0.110 |
|  | Alireza Sadeghi, 2019 | 6 | 3998/ 490255 | dose-response | 1.02 (0.98-1.05) | 1.01 (0.99-1.03) | 0.307 | 0.230 |
| Esophageal adenocarcinoma | Kondwani Joseph Banda, 2020 | 7 | 1358/ 6585 | high vs. low | 0.60 (0.45-0.79) | 0.64 (0.52-0.77) | 2.820×10^-4^ | 4.281×10^-6^ |
|  | Fei Xuan, 2020 | 10 | 1798/ 9459 | high vs. low | 0.57 (0.42-0.78) | 0.64 (0.54-0.77) | 3.788×10^-4^ | 7.549×10^-7^ |
| Esophageal cancer | Fei Xuan, 2020 | 13 | 2833/ 15216 | high vs. low | 0.63 (0.50-0.78) | 0.69 (0.61-0.79) | 2.361×10^-5^ | 2.380×10^-8^ |
|  | Kondwani-Joseph Banda, 2020 | 11 | 2576/ 12559 | high vs. low | 0.62 (0.50-0.77) | 0.67 (0.58-0.77) | 1.429×10^-5^ | 1.010×10^-8^ |
|  | Xianlei Cai,2019 | 9 | 1842/ 440440 | high vs. low | 0.75 (0.53-1.06) | 0.74 (0.62-0.89) | 0.099 | 0.001 |
| Esophageal squamous cell carcinoma | Kondwani-Joseph Banda, 2020 | 8 | 1218/ 5974 | high vs. low | 0.63 (0.45-0.90) | 0.70 (0.57-0.86) | 0.012 | 4.871×10^-4^ |
|  | Fei Xuan, 2020 | 7 | 969/ 5298 | high vs. low | 0.67 (0.45-0.98) | 0.75 (0.60-0.92) | 0.037 | 0.007 |
| Gastric cancer | Yao Ye, 2016 | 22 | 5905/ 83612 | high vs. low | 1.17 (0.91-1.50) | 1.14 (1.03-1.27) | 0.212 | 0.011 |
|  | Xianlei Cai, 2019 | 12 | 2355/ 111631 | high vs. low | 0.84 (0.58-1.22) | 0.96 (0.83-1.12) | 0.368 | 0.607 |
| Liver cancer | Xianlei Cai, 2019 | 6 | 674/ 655527 | high vs. low | 1.04 (0.83-1.30) | 1.04 (0.83-1.30) | 0.720 | 0.720 |
| Pancreatic cancer | Xianlei Cai, 2019 | 9 | 1967/ 773654 | high vs. low | 1.03 (0.87-1.22) | 1.03 (0.89-1.19) | 0.758 | 0.705 |
|  | D. Aune, 2012 (b) | 9 | 3202/ 1112404 | high vs. low | 1.00 (0.86-1.15) | 1.00 (0.88-1.14) | 0.962 | 0.999 |
|  | D. Aune, 2012 (b) | 9 | 3202/ 1112404 | dose-response | 0.97 (0.81-1.16) | 0.99 (0.86-1.14) | 0.713 | 0.857 |

Supplementary table 5. *Continued*

| Outcomes | Individual study | No. of primary studies | No. of  cases/ participants | Comparison | Summary relative risk (95% CI) | | Random  *P* value† | Fixed  *P* value‡ |
| --- | --- | --- | --- | --- | --- | --- | --- | --- |
|  |  |  |  |  | Random effects | Fixed effects |  |  |
| Prostate cancer | Lai lai Fan, 2018 | 22 | 11573/ 98583 | high vs. low | 1.11 (0.98-1.26) | 1.17 (1.10-1.23) | 0.101 | 4.920×10^-8^ |
|  | Long Zhai, 2015 | 18 | 8046/ 84687 | high vs. low | 1.06 (0.93-1.20) | 1.15 (1.08-1.22) | 0.396 | 5.019×10^-8^ |
|  | Rongjiang Wang, 2015 | 13 | 7757/ 76049 | high vs. low | 0.96 (0.81-1.14) | 0.98 (0.87-1.09) | 0.672 | 0.702 |
| Metabolic diseases |  |  |  |  |  |  |  |  |
| Type 2 Diabetes | Dale S. Hardy, 2020 | 14 | 109788/ 440761 | high vs. low | 1.00 (0.92-1.09) | 1.01 (0.95-1.07) | 0.962 | 0.849 |
|  | Greenwood DC, 2013 | 8 | 18403/ 336161 | dose-response | 0.97 (0.90-1.06) | 0.99 (0.95-1.02) | 0.514 | 0.484 |
|  | Amani Alhazmi, 2014 | 8 | 11536/ 488969 | high vs. low, dose-response | 1.11 (1.01-1.22) | 1.13 (1.05-1.21) | 0.035 | 0.001 |
| Metabolic syndrome | Yashu Liu, 2019 | 18 | 69164/ 283150 | high vs. low | 1.25 (1.15-1.37) | 1.24 (1.18-1.29) | 5.262×10^-7^ | 2.477×10^-21^ |
|  | Yashu Liu, 2019 | 10 | 12081/ 45729 | dose-response | 1.02 (1.00-1.05) | 1.01 (1.00-1.02) | 0.023 | 4.523×10^-4^ |
| Digestive system outcomes |  |  |  |  |  |  |  |  |
| Ulcerative colitis | Fan Wang, 2016 | 5 | 540/ 2075 | dose-response | 1.01 (0.99-1.02) | 1.00 (0.99-1.01) | 0.440 | 0.423 |
| Crohn’s disease | Lirong Zeng, 2017 | 4 | 388/ 1344 | dose-response | 0.99 (0.98-1.00) | 0.99 (0.98-1.00) | 0.166 | 0.166 |
| Inflammatory bowel diseases | Zhongqin Jin, 2018 | 15 | 1361/ 332202 | high vs. low | 1.09 (0.82-1.46) | 1.08 (0.93-1.26) | 0.555 | 0.315 |
| Other outcomes |  |  |  |  |  |  |  |  |
| coronary heart disease | Geoffrey Livesey, 2019 | 6 | 2507/ 228209 | dose-response | 1.65 (1.19-2.29) | 1.50 (1.21-1.85) | 0.002 | 1.840×10^-4^ |
|  | Dale S. Hardy, 2020 | 11 | 15316/ 464491 | high vs. low | 1.08 (1.00-1.16) | 1.07 (1.01-1.13) | 0.045 | 0.016 |
| Stroke | Geoffrey Livesey, 2019 | 8 | 7283/ 394020 | high vs. low | 1.11 (0.94-1.31) | 1.11 (0.95-1.30) | 0.228 | 0.197 |
| Parkinson’s disease | Aimin Wang, 2015 | 8 | 1482/ 232869 | high vs. low | 1.24 (1.05-1.48) | 1.24 (1.05-1.48) | 0.014 | 0.014 |
| Bone fracture | H.Mozaffari, 2020 | 5 | 1635/ 38828 | high vs. low | 1.24 (0.84-1.83) | 0.96 (0.82-1.13) | 0.276 | 0.645 |
|  | H.Mozaffari, 2020 | 6 | 1765/ 41341 | dose-response | 1.00 (0.94-1.05) | 1.00 (0.99-1.01) | 0.896 | 0.661 |

CI, confidence interval

* Specific-cause mortality included hemorrhagic and ischemic stroke mortality

§ Specific-cause mortality included Type 2 Diabetes mortality, coronary heart disease mortality, and stroke mortality

† *P* value of summary random effects estimate

‡ *P* value of summary fixed effects estimate

All statistical tests were two-sided.

Supplementary table 6. Credibility assessment of evidence for all meta-analyses reporting association of dietary carbohydrate intake relation to multiple outcomes.

| Outcomes | Individual study | Features used for credibility assessment of evidence | | | | | | Evidence classification |
| --- | --- | --- | --- | --- | --- | --- | --- | --- |
|  |  | Sample size† | Statistical significance‡ | Largest study  Significance | 95% prediction interval | Estimate of heterogeneity# | Small-study effect/ excess significant bias |  |
| Mortality |  |  |  |  |  |  |  |  |
| All-cause mortality | Sara B Seidelmann, 2018 | > 1000 | < 10^-3^ | > 0.05 | Including the null value | Large | Small-study effects | Suggestive |
| Stroke and specific-cause mortality* | Xianlei Cai, 2015 | > 1000 | > 0.05 | > 0.05 | Including the null value | Not large | Neither | No association |
| All-cause and specific-cause mortality§ | Dale S. Hardy, 2020 | > 1000 | > 0.05 | > 0.05 | Including the null value | Not large | Neither | No association |
| Cancer |  |  |  |  |  |  |  |  |
| Breast cancer | Sabrina Schlesinger, 2017 | > 1000 | > 0.05 | > 0.05 | Including the null value | Large | Neither | No association |
| Colorectal cancer | D. Aune, 2012 (a) | > 1000 | > 0.05 | > 0.05 | Including the null value | Not large | Neither | No association |
|  | D. Aune, 2012 (a) | > 1000 | > 0.05 | < 0.05 | Including the null value | Large | Neither | No association |
|  | Xianlei Cai, 2019 | > 1000 | > 0.05 | > 0.05 | Including the null value | Not large | Neither | No association |
|  | Jian Huang, 2017 | > 1000 | > 0.05 | > 0.05 | Including the null value | Large | Small-study effects | No association |
| Digestive system cancers | Xianlei Cai, 2019 | > 1000 | > 0.05 | > 0.05 | Including the null value | Not large | Neither | No association |
|  | Xianlei Cai, 2019 | > 1000 | > 0.05 | > 0.05 | Including the null value | Very large | Neither | No association |
| Endometrial cancer | Alireza Sadeghi, 2019 | > 1000 | > 0.05 | > 0.05 | Including the null value | Large | Neither | No association |
|  | Alireza Sadeghi, 2019 | > 1000 | > 0.05 | > 0.05 | Including the null value | Large | Neither | No association |
| Esophageal adenocarcinoma | Kondwani Joseph Banda, 2020 | > 1000 | < 10^-3^ | > 0.05 | Including the null value | Not large | Neither | Suggestive |
|  | Fei Xuan, 2020 | > 1000 | < 10^-3^ | > 0.05 | Including the null value | Large | Neither | Suggestive |
| Esophageal cancer | Fei Xuan, 2020 | > 1000 | < 10^-4^ | > 0.05 | Including the null value | Large | Small-study effects | Suggestive |
|  | Kondwani Joseph Banda, 2020 | > 1000 | < 10^-4^ | > 0.05 | Including the null value | Large | Small-study effects | Suggestive |
|  | Xianlei Cai,2019 | > 1000 | > 0.05 | > 0.05 | Including the null value | Large | Neither | No association |
| Esophageal squamous cell carcinoma | Kondwani Joseph Banda, 2020 | > 1000 | < 0.05 | > 0.05 | Including the null value | Large | Neither | Weak |
|  | Fei Xuan, 2020 | < 1000 | < 0.05 | > 0.05 | Including the null value | Large | Neither | Weak |

Supplementary table 6. *Continued*

| Outcomes | Individual study | Features used for credibility assessment of evidence | | | | | | Evidence classification |
| --- | --- | --- | --- | --- | --- | --- | --- | --- |
|  |  | Sample size† | Statistical significance‡ | Largest study  Significance | 95% prediction interval | Estimate of heterogeneity# | Small-study effect/ excess significant bias |  |
| Gastric cancer | Yao Ye, 2016 | > 1000 | > 0.05 | < 0.05 | Including the null value | Very large | Neither | No association |
|  | Xianlei Cai, 2019 | > 1000 | > 0.05 | > 0.05 | Including the null value | Very large | Neither | No association |
| Liver cancer | Xianlei Cai, 2019 | < 1000 | > 0.05 | > 0.05 | Including the null value | Not large | Neither | No association |
| Pancreatic cancer | Xianlei Cai, 2019 | > 1000 | > 0.05 | > 0.05 | Including the null value | Not large | Neither | No association |
|  | D. Aune, 2012 (b) | > 1000 | > 0.05 | > 0.05 | Including the null value | Not large | Neither | No association |
|  | D. Aune, 2012 (b) | > 1000 | > 0.05 | > 0.05 | Including the null value | Not large | Neither | No association |
| Prostate cancer | Lai lai Fan, 2018 | > 1000 | > 0.05 | < 0.05 | Including the null value | Large | Neither | No association |
|  | Long Zhai, 2015 | > 1000 | > 0.05 | < 0.05 | Including the null value | Not large | Neither | No association |
|  | Rongjiang Wang, 2015 | > 1000 | > 0.05 | > 0.05 | Including the null value | Large | Neither | No association |
| Metabolic diseases |  |  |  |  |  |  |  |  |
| Type 2 Diabetes | Dale S. Hardy, 2020 | > 1000 | > 0.05 | > 0.05 | Including the null value | Not large | Neither | No association |
|  | Greenwood DC, 2013 | > 1000 | > 0.05 | < 0.05 | Including the null value | Very large | Neither | No association |
|  | Amani Alhazmi, 2014 | > 1000 | < 0.05 | < 0.05 | Including the null value | Not large | Neither | Weak |
| Metabolic syndrome | Yashu Liu, 2019 | > 1000 | < 10^-6^ | < 0.05 | Including the null value | Large | Excess significance bias | Highly suggestive |
|  | Yashu Liu, 2019 | > 1000 | < 0.05 | > 0.05 | Including the null value | Very large | Neither | Weak |
| Digestive system outcomes |  |  |  |  |  |  |  |  |
| Ulcerative colitis | Fan Wang, 2016 | < 1000 | > 0.05 | > 0.05 | Including the null value | Not large | Neither | No association |
| Crohn’s disease | Lirong Zeng, 2017 | < 1000 | > 0.05 | > 0.05 | Including the null value | Not large | Neither | No association |
| Inflammatory bowel diseases | Zhongqin Jin, 2018 | > 1000 | > 0.05 | > 0.05 | Including the null value | Not large | Neither | No association |
| Other outcomes |  |  |  |  |  |  |  |  |
| coronary heart disease | Geoffrey Livesey, 2019 | > 1000 | < 0.05 | > 0.05 | Including the null value | Not large | Neither | Weak |
|  | Dale S. Hardy, 2020 | > 1000 | < 0.05 | > 0.05 | Including the null value | Not large | Neither | Weak |

Supplementary table 6. *Continued*

| Outcomes | Individual study | Features used for credibility assessment of evidence | | | | | | Evidence classification |
| --- | --- | --- | --- | --- | --- | --- | --- | --- |
|  |  | Sample size† | Statistical significance‡ | Largest study  Significance | 95% prediction interval | Estimate of heterogeneity# | Small-study effect/ excess significant bias |  |
| Stroke | Geoffrey Livesey, 2019 | > 1000 | > 0.05 | > 0.05 | Including the null value | Not large | Neither | No association |
| Parkinson’s disease | Aimin Wang, 2015 | > 1000 | < 0.05 | > 0.05 | Including the null value | Not large | Neither | Weak |
| Bone fracture | H.Mozaffari, 2020 | > 1000 | > 0.05 | > 0.05 | Including the null value | Large | Neither | No association |
|  | H.Mozaffari, 2020 | > 1000 | > 0.05 | > 0.05 | Including the null value | Large | Neither | No association |

* Specific-cause mortality included hemorrhagic and ischemic stroke mortality

§ Specific-cause mortality included Type 2 Diabetes mortality, CHD mortality, and stroke mortality

† Number of cases

‡ *P* value under the random-effects model

# Heterogeneity was categorized as not large (I²< 50%), large (I ²≥5 0% but I² ≤ 75%), and very large (I² > 75%).

Supplementary table 7. The detailed results of methodological quality assessment.

| Study | AMSTAR | | | | | | | | | | | Total points | Quality* |
| --- | --- | --- | --- | --- | --- | --- | --- | --- | --- | --- | --- | --- | --- |
|  | 1-item | 2-item | 3-item | 4-item | 5-item | 6-item | 7-item | 8-item | 9-item | 10-item | 11-item |  |  |
| Yao Ye 2016 | CA | CA | Yes | No | No | Yes | Yes | Yes | Yes | Yes | Yes | 7 | high quality |
| Kondwani Joseph Banda 2020 | CA | Yes | Yes | No | No | Yes | Yes | Yes | Yes | Yes | Yes | 8 | high quality |
| Alireza Sadeghi 2019 | CA | Yes | Yes | No | CA | Yes | Yes | Yes | Yes | Yes | Yes | 8 | high quality |
| Lai lai Fan 2018 | CA | Yes | Yes | CA | CA | Yes | Yes | Yes | Yes | Yes | CA | 7 | high quality |
| Sabrina Schlesinger 2017 | Yes | CA | Yes | CA | No | Yes | CA | Yes | CA | Yes | Yes | 6 | moderate quality |
| D. Aune 2012 (a) | Yes | Yes | Yes | CA | No | Yes | CA | Yes | Yes | Yes | CA | 7 | moderate quality |
| Long Zhai 2015 | CA | Yes | No | CA | CA | Yes | CA | Yes | Yes | Yes | Yes | 6 | moderate quality |
| Fei Xuan 2020 | CA | Yes | No | CA | No | Yes | Yes | Yes | Yes | Yes | Yes | 7 | moderate quality |
| Xianlei Cai 2019 | Yes | Yes | Yes | No | No | Yes | Yes | Yes | Yes | Yes | Yes | 9 | high quality |
| D. Aune 2012 (b) | Yes | CA | No | CA | No | Yes | Yes | Yes | Yes | Yes | Yes | 7 | moderate quality |
| Rongjiang Wang 2015 | CA | CA | Yes | CA | CA | Yes | Yes | Yes | Yes | Yes | Yes | 7 | moderate quality |
| Jian Huang 2017 | CA | Yes | Yes | No | No | Yes | CA | Yes | Yes | Yes | Yes | 7 | moderate quality |
| Geoffrey Livesey 2019 | Yes | CA | CA | CA | No | Yes | Yes | Yes | Yes | Yes | Yes | 7 | moderate quality |
| Dale S. Hardy 2020 | CA | CA | CA | No | No | Yes | Yes | Yes | Yes | Yes | Yes | 7 | moderate quality |
| Darren C. G Reenwood 2013 | Yes | Yes | Yes | No | No | Yes | Yes | Yes | Yes | Yes | Yes | 9 | high quality |
| Amani Alhazmi 2013 | Yes | Yes | Yes | Yes | CA | Yes | Yes | Yes | Yes | Yes | Yes | 10 | high quality |
| Yashu Liu 2019 | Yes | Yes | Yes | CA | Yes | Yes | Yes | Yes | Yes | Yes | Yes | 10 | high quality |
| Aimin Wang 2015 | CA | Yes | Yes | CA | CA | Yes | CA | Yes | Yes | Yes | Yes | 7 | high quality |
| Xianlei Cai 2015 | CA | CA | Yes | No | CA | Yes | Yes | Yes | Yes | Yes | Yes | 7 | moderate quality |
| Fan Wang 2016 | CA | Yes | Yes | No | CA | Yes | Yes | Yes | Yes | Yes | Yes | 8 | moderate quality |
| Lirong Zeng 2017 | CA | Yes | Yes | No | CA | Yes | Yes | Yes | Yes | Yes | CA | 7 | moderate quality |
| Zhongqin Jin 2018 | CA | Yes | CA | No | CA | Yes | Yes | Yes | Yes | Yes | Yes | 7 | moderate quality |
| Sara B Seidelmann 2018 | CA | CA | No | CA | CA | Yes | Yes | Yes | Yes | CA | Yes | 5 | moderate quality |
| H. Mozaffari 2020 | CA | Yes | Yes | CA | CA | Yes | Yes | Yes | Yes | Yes | Yes | 8 | high quality |

AMSTAR, a measurement tool to assess the methodological quality of systematic review; CA, cannot answer

*High quality: at least 8 points; moderate quality: 4-7 points; low quality: 3 points or less.

**Supplementary Figure legends**

Supplementary Figure 1. Map of dietary carbohydrate intake related outcomes: percentage of outcomes per outcome category

Supplementary Figure 2. The detailed results of methodological quality assessment.
