# Supplementary figures and images for: Dietary Carbohydrate and Diverse Health Outcomes: Umbrella Review of 30 Systematic Reviews and Meta-Analyses of 281 Observational Studies
Source: Front Nutr. 2021 Apr 29;8:670411. doi: 10.3389/fnut.2021.670411 (PMC8116488; doi:10.3389/fnut.2021.670411)

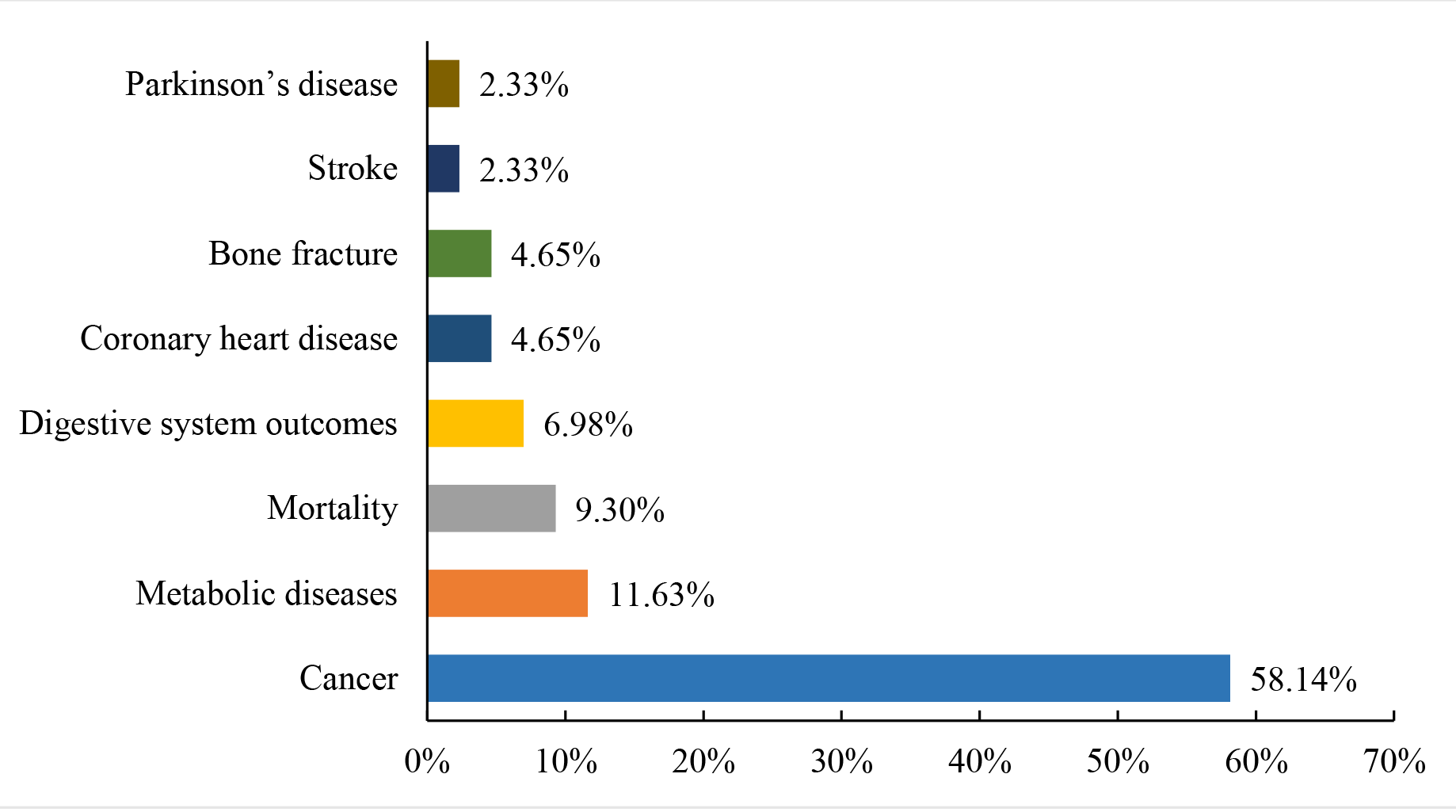

Supplement: Supplementary file 2 [file Image_1.TIF]

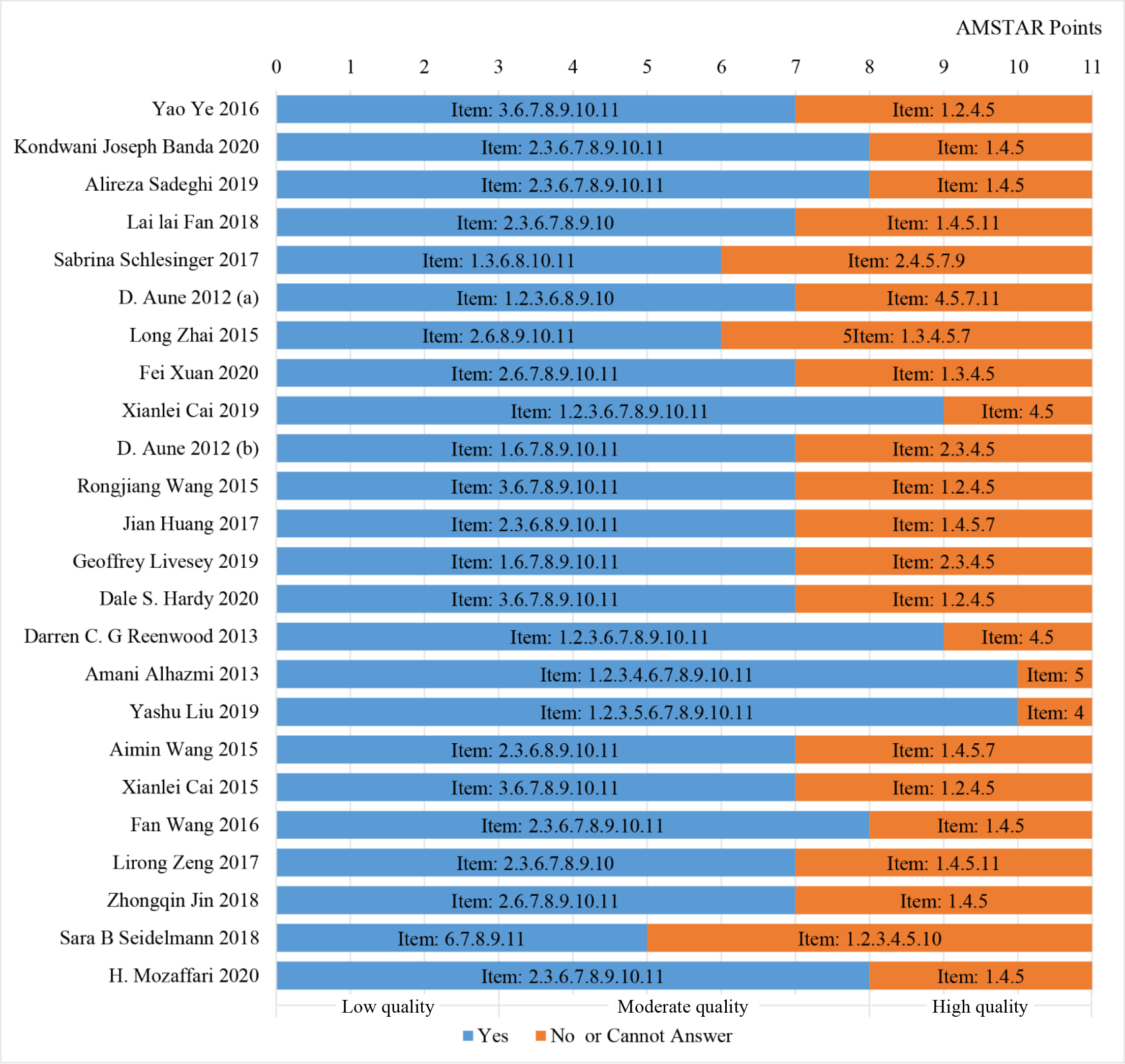

Supplement: Supplementary file 3 [file Image_2.TIF]
